# Secular trends in incidence and mortality of cervical cancer in India and its states, 1990-2019: data from the Global Burden of Disease 2019 Study

**DOI:** 10.1186/s12885-022-09232-w

**Published:** 2022-02-07

**Authors:** Mayank Singh, Ravi Prakash Jha, Neha Shri, Krittika Bhattacharyya, Priyanka Patel, Deepak Dhamnetiya

**Affiliations:** 1 Department of Fertility Studies, International Institute for Population Sciences (IIPS), Mumbai, 400088 India; 2 Department of Community Medicine, Dr. Baba Saheb Ambedkar Medical College & Hospital, Delhi, 110085 India; 3 International Institute for Population Sciences (IIPS), Mumbai, 400088 India; 4 Department of Statistics, University of Calcutta, Kolkata, 700019 India; 5 Department of Development Studies, International Institute for Population Sciences (IIPS), Mumbai, 400088 India

**Keywords:** Cervical cancer, Incidence, Mortality, Trend, Joinpoint regression analysis, India, GBD

## Abstract

**Background:**

Cervical cancer is the fourth most common cancer that occurs to women worldwide. This study aims to assess trends in incidence and mortality of cervical cancer in India and its states over past three decades for tracking the progress of strategies for the prevention and control of cervical cancer.

**Methods:**

Data on cervical cancer incidence and mortality from 1990 to 2019 for India and its states were extracted from Global Burden of Disease study and were utilized for the analysis. Spatial and rank map has been used to see the changes in incidence and mortality of cervical cancer in different Indian states. Further, joinpoint regression analysis is applied to determine the magnitude of the time trends in the age standardized incidence and mortality rates of cervical cancer. We obtained the average annual percent change (AAPC) and corresponding 95% confidence intervals (CI) for each state.

**Results:**

Overall, from 1990 to 2019 Jharkhand (Incidence: -50.22%; Mortality: -56.16%) recorded the highest percentage decrement in cervical cancer incidence and mortality followed by the Himachal Pradesh (Incidence: -48.34%; Mortality: -53.37%). Tamilnadu (1^st^ rank), Jammu & Kashmir and Ladakh (32^nd^ rank) maintained the same rank over the period of three decade for age standardized cervical cancer incidence and mortality. The regression model showed a significant declining trend in India between 1990 and 2019 for age standardized incidence rate (AAPC: −0.82; 95%CI: −1.39 to −0.25; *p* < 0.05) with highest decline in the period 1998-2005 (AAPC: −3.22; 95%CI: −3.83 to −2.59; *p* < 0.05). Similarly, a significant declining trend was observed in the age standardized mortality rate of India between 1990 and 2019(AAPC: −1.35; 95%CI: −1.96 to −0.75; *p* < 0.05) with highest decline in the period 1998-2005 (AAPC: −3.52; 95%CI: −4.17 to −2.86; *p* < 0.05).

**Conclusion:**

Though the incidence and mortality of cervical cancer declined over past three decades but it is still a major public health problem in India. Information, education and communication activities for girls, boys, parents and community for the prevention and control of cervical cancer should be provided throughout the country.

**Supplementary Information:**

The online version contains supplementary material available at 10.1186/s12885-022-09232-w.

## Background

Cervical cancer affects the lowermost part of a women’s uterus, called the “cervix”. Worldwide, cervical cancer is the fourth most common cancer in women [1]. Despite being a highly preventable cancer, in the year 2020, 604,127women were reported with cervical cancer, and 341,831women died from the disease globally [2]. The maximum proportion of cervical cancer cases was reported from Asia (58.2%) and the minimum from the Northern America region (2.5%). Recent estimates for 2018 show that annually 569847 new cervical cancer cases were diagnosed worldwide. Around 2785 million women are at risk of getting cervical cancer, and approximately four-fifths of them are from less developed regions. Globally, the age-standardized incidence and mortality rates are found to be 13.1 and 6.9 per 100,000 women [3]. However, these rates are quite higher among Indian women in comparison to global estimates. In India, the age-standardized incidence rate is 14.7 per 100,000 women, and the age-standardized mortality rate is 9.2 per 100,000 women [3].

Cervical cancer is associated with sexual behaviours such as poor genital hygiene, early age of marriage, multiple sexual partners, repeated pregnancies [4]. Cofactors such as long-term contraceptive use, smoking are associated with HPV infection. Specific women populations, such as women in correctional facilities, are at higher risk for cervical cancer than women in the general population [5]. Around 453 million Indian women aged 15 years and above are at risk of developing cancer [3]. Estimates from the HPV information centre show that 96922 women are diagnosed with cervical cancer every year, and 60078 of them die of this disease in the country. Current data indicates that that cervical cancer is the second most common cancer among females in the country. Low age at marriage, early age at first intercourse, higher parity raises the risk of HPV acquisition among Indian women [6]. Although the burden of cervical cancer is increasing largely in the country, deaths can be prevented if it is screened at early stages [7]. Cervical cancer mostly affects women from rural areas with poor socioeconomic status [8]. Poor screening is associated with poverty. Lack of screening and treatment has been identified as a factor leading to the development of invasive cancer, which leads to death [9, 10]. Although the improvement in the living standard and awareness among women has resulted in a decline in the incidence of cervical cancer in the country, the situation is alarming in the rural settings where the majorities of women are illiterate and have poor hygienic conditions.

Additionally, access to medical facilities and poor socioeconomic status contribute to the spread of carcinoma cervix in rural areas. As India is on the way to universalizing the national level screening programme of cervical cancer, it is crucial to investigate the areas vulnerable to poor screening and trends and patterns in its prevalence. Screening and associated social determinants are vital to understanding the need for intervention in a heterogeneous population like India. There has been substantial progress in primary prevention strategies, and it certainly affected incidence and mortality due to cervical cancer. However, screening for precancerous and cancerous cervical lesions among women over 30 years will be critical in developing countries like India to ensure that women receive appropriate diagnostic and treatment services. This study aims to assess trends in incidence and mortality of cervical cancer in India and its states over the past three decades to track the progress of strategies for preventing and controlling cervical cancer.

## Material and methods

We have extracted data on cervical cancer incidence and mortality from 1990 to 2019 from the Global Burden of Disease 2019 study. The case definition includes cervical cancer having ICD-10 codes C53, C53.0, C53.1, C53.3, C53.4, C53.8, C53.9, D06, D06.0, D06.1, D06.7, D06.9, D26.0. Data for the incidence and Death rate of cervical cancer for India were extracted from an online tool produced by the IHME, which is publicly available called the GHDx (Global Health Data Exchange) query tool (http://ghdx.healthdata.org/gbd-results-tool) [11]. The state-level data on the burden of cervical cancer is extracted from the GBD India Compare tool (https://vizhub.healthdata.org/gbd-compare/india) [12]. The key sources of data that GBD used to model the cause of death due to cervical cancer in India includes cancer incidence in five continents by the International Agency for Research on Cancer, International Association of Cancer Registries, Population-based cancer registries of India and various states, medical certification of cause of deaths of the country and various states, vital statistics, other surveys on the cause of death and published scientific articles [13].

A spatial map of age-standardized incidence and mortality rate has been used at 10-year intervals to understand the trends in cervical cancer incidence and mortality rate over time and space. Further, state-wise changes in cervical cancer incidence and mortality rank have been shown through the graph. Joinpoint regression analysis has been applied to compute the magnitude of the time trends in the age-standardized incidence and mortality rates of Cervical Cancer, the Average Annual Percent Change (AAPC) and the corresponding 95% Confidence Interval (CI). By using rates as inputs, the joinpoint regression method identifies the year(s) when a trend change is evident and calculates the annual percentage change (APC) in rates between these trend-change points.

To estimate the APC, the following model is used:

log(*Y*_*x*_) = *b*_0_ + *b*_1_*x* , where log (Yx) is the natural logarithm of the rate in year x.

Then, the APC from year *x* to year *x* + *1* is:

$$APC=\frac{e^{b_0+{b}_1\left(x+1\right)}-{e}^{b_0+{b}_1x}}{e^{b_0+{b}_1x}}\ast 100=\left({e}^{b_1}-1\right)\ast 100$$

When no joint point is detected over the period, then APC and AAPC will be the same. However, In case of any trend changes over the period, the whole period is segmented by the points with trend change [14]. AAPC was calculated as a geometrically weighted average of various annual percent change (APC) values from the regression analysis [15]. For the whole range of our study periods, the average APC (AAPC) is computed using the best model with a maximum of 5 joinpoints pertaining to 6 segments. Joinpoint regression analysis is performed using ‘Joinpoint Regression Program’ software (version 4.9.0.0) provided by the Surveillance Research Program of the US National Cancer Institute.

## Results

### Incidence of cervical cancer among women in India in the period 1990-2019

**Fig. 1:**
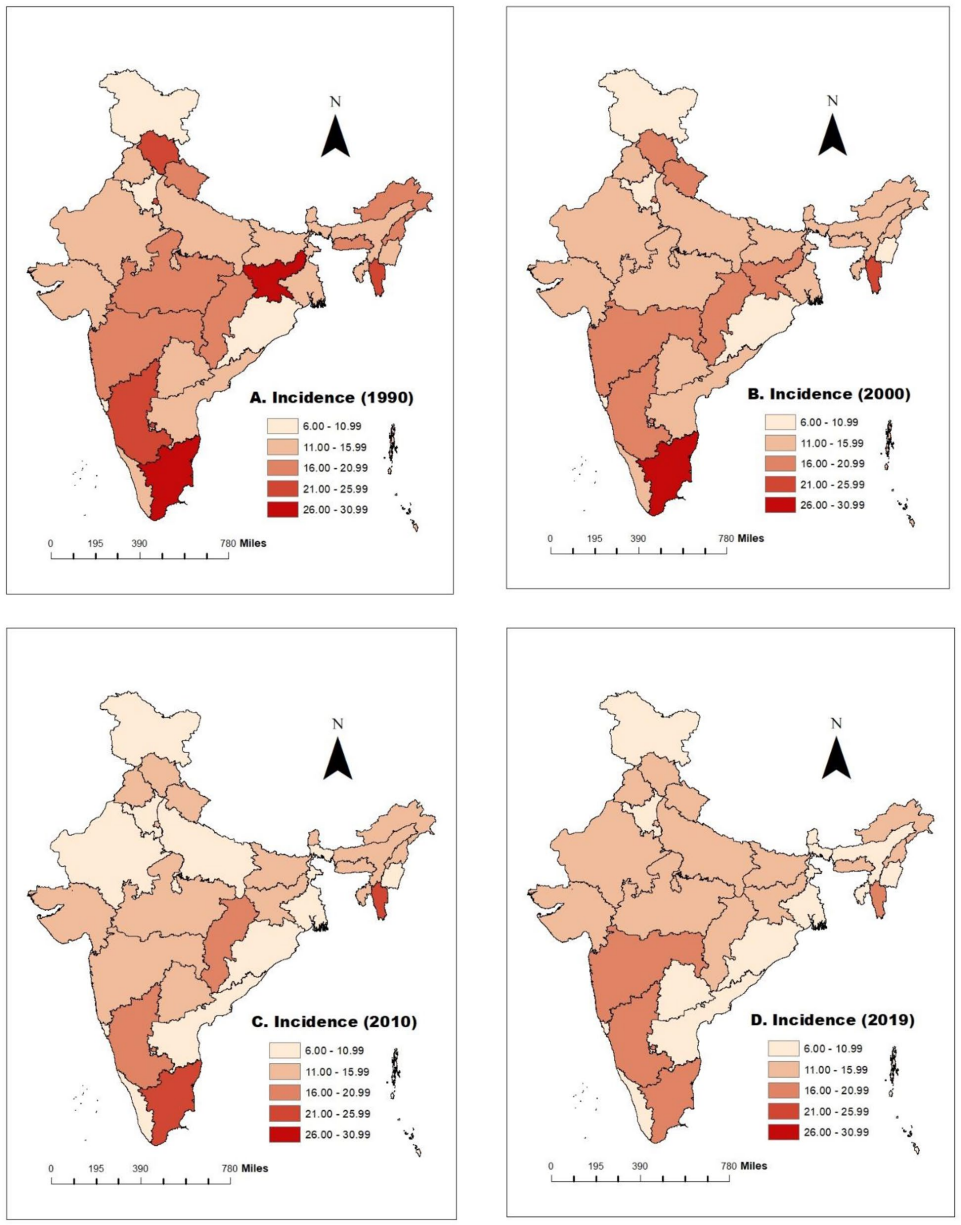
Incidence of cervical cancer from 1990 to 2019 per 100,000 women in India. **A** Incidence of cervical cancer per 100,000 women in 1990. **B** Incidence of cervical cancer per 100,000 women in 2000. **C** Incidence of cervical cancer per 100,000 women in 2010. **D** Incidence of cervical cancer per 100,000 women in 2019

Figure 1 represents the state-wise incidence of cervical cancer among women from 1990 to 2019. As evident from the maps (Fig. 1), decrement of incidence is not uniform over time across the states. Some of the states like Jharkhand (-28.47%) and Gujarat (-23.27%) shows the highest percentage decline in the incidence of cervical cancer in the period 1990-2000. Further in the next decennial (2000-2010), Himachal Pradesh (-30.87%) followed by West Bengal (-28.56%) takes the credit for the highest percentage decrement in cervical cancer incidence. Overall, from 1990 to 2019, Jharkhand (-50.22%) recorded the highest percentage decrement, followed by Himachal Pradesh (-48.34%) (Supplementary Table 1). Jammu & Kashmir report the lowest incidence (7.24 in 1990, 6.31 in 2000, 6.21 in 2010 and 6.13 in 2019), whereas Tamilnadu has the highest incidence (30.92 in 1990, 28.26 in 2000, 21.58 in 2010 and 19.91 in 2019) of cervical cancer from 1990 to 2019.

### Mortality due to cervical cancer among women in India in the period 1990-2019

**Fig. 2:**
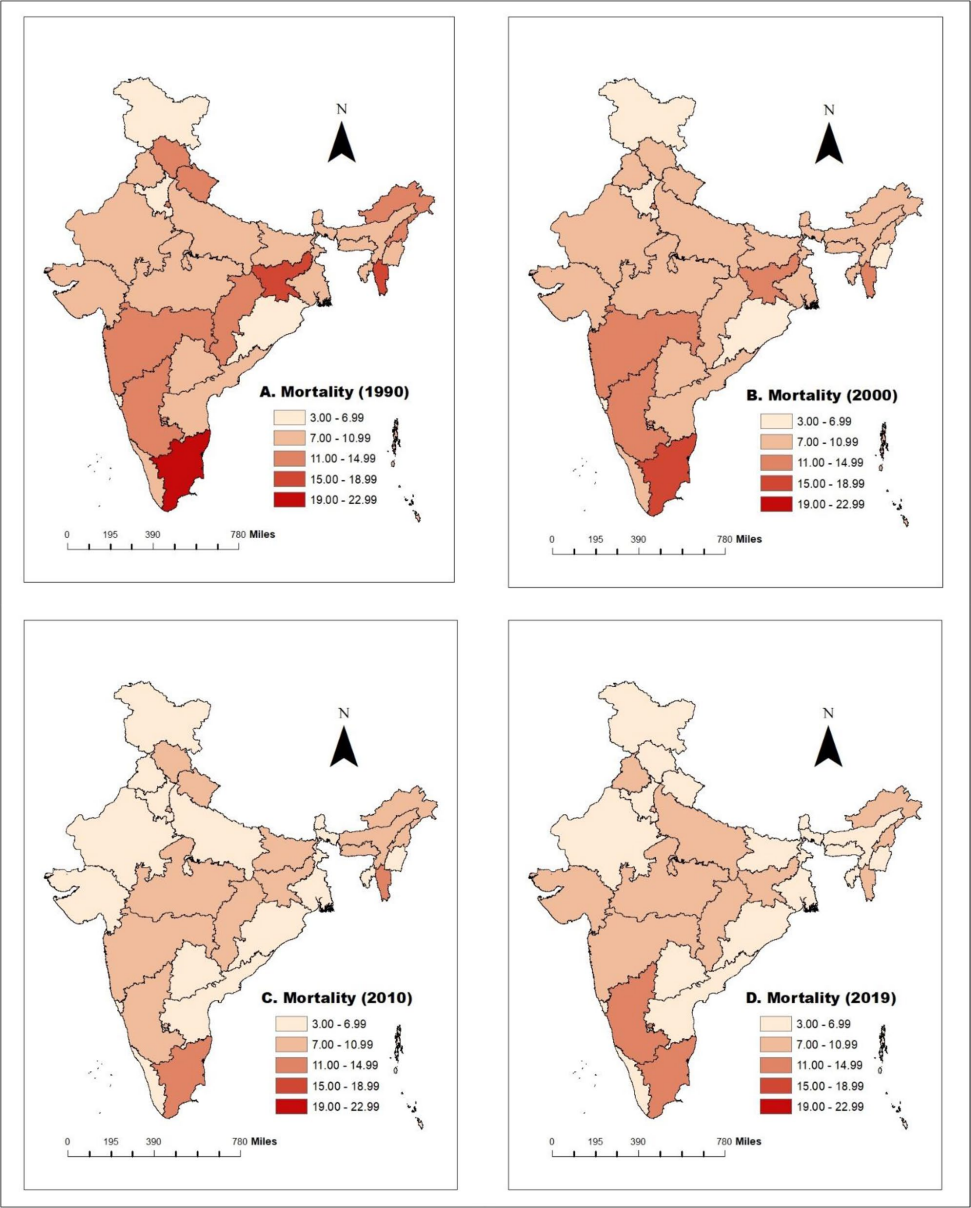
Mortality of cervical cancer from 1990 to 2019 per 100,000 women in India. **A** Mortality of cervical cancer per 100,000 women in 1990. **B** Mortality of cervical cancer per 100,000 women in 2000. **C** Mortality of cervical cancer per 100,000 women in 2010. **D** Mortality of cervical cancer per 100,000 women in 2019

Figure 2 represents the state-wise mortality of cervical cancer among women for 1990 - 2019. Figure 2 (A, B, C & D) represents that cervical cancer mortality has decreased over time while the decrement in mortality is not uniform over time. Jammu & Kashmir have the lowest mortality level (4.59 per 100,000 women in 1990, 3.93 per 100,000 women in 2000, 3.57 per 100,000 women in 2010 and 3.38 per 100,000 women in 2019) whereas Tamilnadu records the highest mortality due to cervical cancer(20.73 per 100,000 women in 1990, 18.62per 100,000 women in 2000, 13.53 per 100,000 women in 2010 and 11.56per 100,000 women in 2019) from 1990 to 2019. Maps in Fig. 2 show that decrement in the incidence is not uniform across the states over time. Some of the states like Jharkhand (-30.42%) and Gujarat (-27.00%) show the highest percentage decline in mortality due to cervical cancer in the period 1990-2000. Further in the next decennial (2000-2010), West Bengal (-33.83%) followed by Himachal Pradesh (-33.02%) have the highest percentage decrement in cervical cancer mortality. Overall, from 1990 to 2019, Jharkhand (-56.16%) recorded the highest percentage decrement, followed by the Himachal Pradesh (-53.37%) (Supplementary Table 2).

### Age-standardized cervical cancer incidence transition in India

**Fig. 3:**
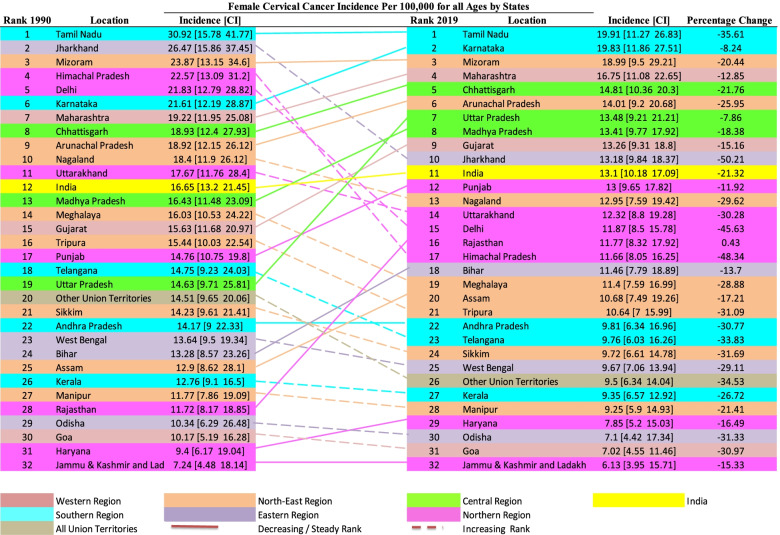
Ranks of Age-Standardized cervical cancer incidence rate per 100,000 women population for all ages in 1990 and 2019 in India

Figure 3 indicates the overall variation in the ranks of age-standardized cervical cancer incidence over the three decades (1990 to 2019). Among all the states and Union territories, the percentage change is negative, i.e. all the states and union territories have witnessed a decline in the incidence rate over time. Percentage decrement in incidence is not uniform across the states, which further leads to variation in states rank. Over the thirty years, ranks of only four states, namely Tamil Nadu (1^st^ rank, -35.61 percentage change), Mizoram (3^rd^ rank, -20.44 percentage change), Andhra Pradesh (22^nd^ rank, -30.77 percentage change), and Jammu & Kashmir &Ladakh (32^nd^ rank, -15.33 percentage change) have remained same, and the ranks of remaining states have changed. The first rank indicates the highest incidence of cervical cancer, and the last (32^nd^) rank indicates the lowest value in age-standardized cervical cancer incidence. The highest percentage change in age-standardized incidence is found in Jharkhand (-50.21%, 2^nd^ rank in 1990 to 10^th^ rank in 2019) followed by Himachal Pradesh (-48.34%, 4^th^ rank in 1990 to 17^th^ rank in 2019) in contrast to the lowest change in incidence is observed in the state Uttar Pradesh (-7.86%, 19^th^ rank in 1990 to 7^th^ rank in 2019) followed by Karnataka (-8.24%, 6^th^ rank in 1990 to 2^nd^ rank in 2019). Only a single state, namely Rajasthan, have shown an increase in the incidence value over time (0.43%, 28^th^ rank in 1990 to 16^th^ rank in 2019). In 1990, 11 states ranked lower than the national average (India 12^th^ rank, incidence 16.65 per 100000), but in 2019 a total of 10 states rank lower than the national average (India 11^th^ rank, incidence 13.1 per 100000).

Among seven northern states, half of the states (Himachal Pradesh, Delhi, Uttarakhand) have moved upward in their rank and other states like Punjab, Rajasthan, Haryana) have (Jammu & Kashmir and Ladakh) remained the same in their rank. In the country's north-eastern region, two states, i.e. Arunachala Pradesh and Assam ranks, have decreased; however, Mizoram has remained the same in their positional rank and ranks of these five states, i.e. Nagaland, Meghalaya, Tripura, Sikkim, Manipur have increased.

### Age-standardized cervical cancer mortality transition in India

**Fig. 4:**
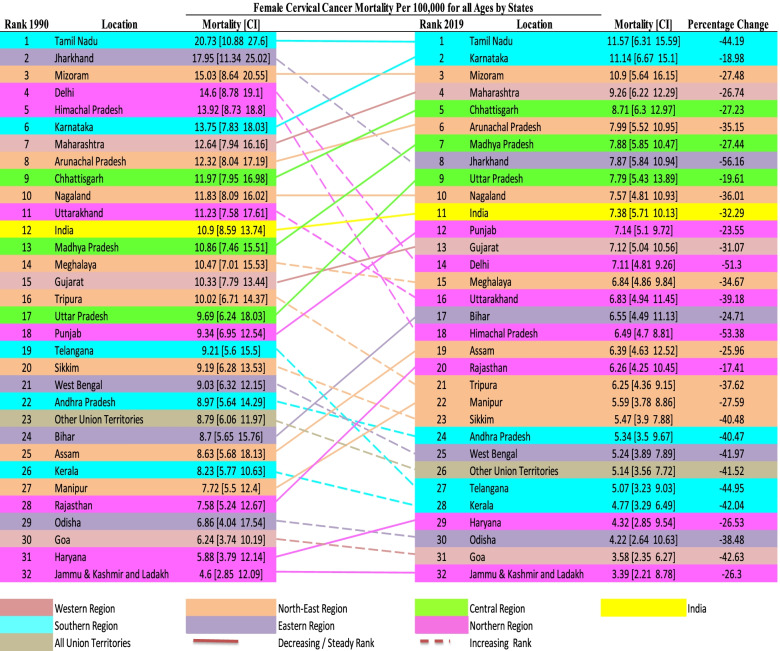
Ranks of age-standardized cervical cancer mortality rate per 100,000 women population for all ages in 1990 and 2019 in India

Figure 4 indicates the variation in ranks of age-standardized cervical cancer mortality among all ages over the three decades (1990 to 2019). The percentage change is negative across all states and Union territories, indicating all the states and union territories have witnessed a decline in mortality over time. The variation in the state ranking indicates that percentage decrement in mortality is not uniform. Overall, Tamil Nadu has performed worst in case of age-standardized cervical cancer incidence 30.92 (CI: 15.78 – 41.77) in 1990 to 19.91 (CI: 11.27 – 26.83) in 2019 and mortality 20.73 (CI: 10.88 – 27.6) in 1990 to 11.57 (CI: 6.31 – 15.59)) per 100,000 females in 2019 both. Whereas Jammu & Kashmir & Ladakh performed well in terms of age-standardized cervical cancer incidence 7.24 (CI: 4.48 – 18.14) in 1990 to 6.13 (CI: 3.95 – 15.71) in 2019 and mortality 4.6 (CI: 2.85 – 12.09) in 1990 to 3.39 (CI: 2.21 – 8.78) per 100,000 females in 2019. Both the highest and lowest performing states have maintained their rank over the period. In the year 1990, 11 states had ranks lower than the national average (India 12^th^ rank, mortality 10.9 per 100000 females). Still, in 2019 only ten states show lower rankings than the national average (India 11^th^ rank, mortality 7.38 per 100000 females).

### Trends in age-standardized cervical cancer incidence and death rates using joinpoint regression analysis across India

**Fig. 5:**
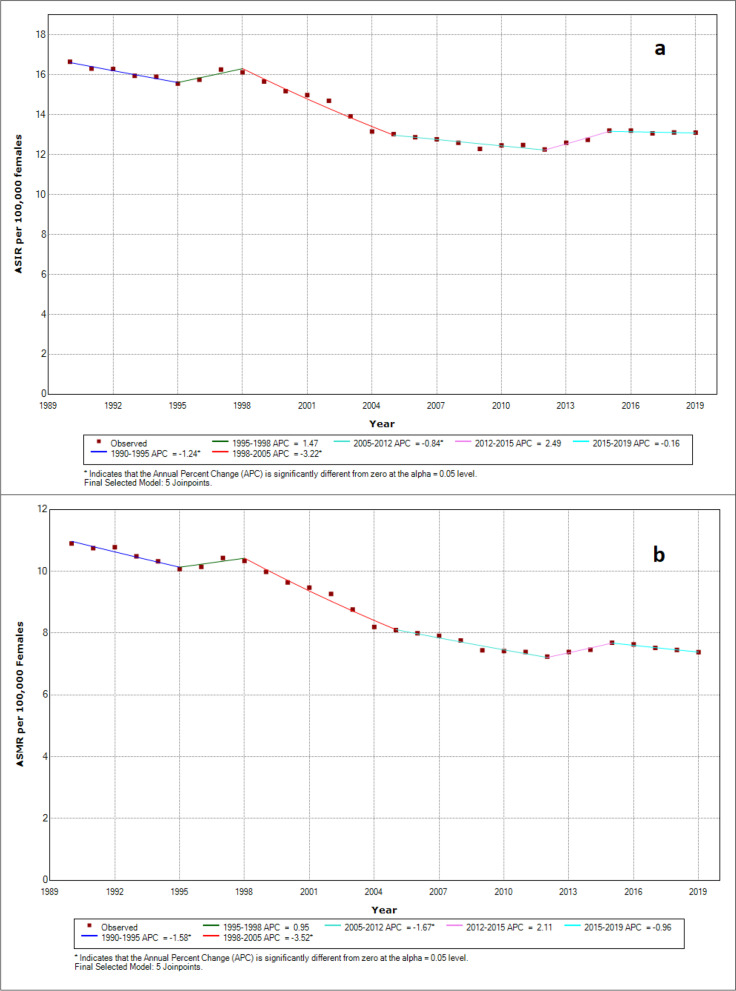
Trends in age-standardized cervical cancer **a**) Incidence and **b**) Death Rates Using Joinpoint Regression Analysis across India

Table 1 shows the APC and AAPC of incidence and mortality due to cervical cancer in India from 1990 to 2019). The regression model shows a significant declining trend in India between 1990 and 2019 for age-standardized incidence rate (AAPC: −0.82; 95%CI: −1.39 to −0.25; *p* < 0.05) with highest decline in the period 1998-2005 (AAPC: −3.22; 95%CI: −3.83 to −2.59; *p* < 0.05) (Table 1, Fig. 5a). Similarly, a significant declining trend has been observed in the age-standardized mortality rate India between 1990 and 2019 (AAPC: −1.35; 95%CI: −1.96 to −0.75; *p* < 0.05) with highest decline in the period 1998-2005 (AAPC: −3.52; 95%CI: −4.17 to −2.86; *p* < 0.05) (Table 1, Fig. 5b).

**Table 1:** Trends in incidence and mortality of cervical cancer in India from 1990 to 2019 using joinpoint regression analysis

Age standardised incidence rate			Age standardised mortality rate		
Segment	Year	APC* (95% C.I.)	Segment	Year	APC* (95% C.I.)
1	1990-1995	-1.24* (-2.07, -0.40)	**1**	1990-1995	-1.58* (-2.46, -0.68)
2	1995-1998	1.47 (-2.31,5.39)	**2**	1995-1998	0.95 (-3.05,5.11)
3	1998-2005	-3.22* (-3.83, -2.59)	**3**	1998-2005	-3.52* (-4.17, -2.86)
4	2005-2012	-0.84* (-1.47, -0.20)	**4**	2005-2012	-1.67* (-2.34, -0.99)
5	2012-2015	2.49 (-1.33,6.45)	**5**	2012-2015	2.11 (-1.94,6.32)
6	2015-2019	-0.16 (-1.35,1.05)	**6**	2015-2019	-0.96 (-2.22,0.31)
AAPC*	1990-2019	-0.82* (-1.39, -0.25)	AAPC*	1990-2019	-1.35* (-1.96, -0.75)

*Note*: *, Indicates that the Annual Percent Change (APC) is significantly different from zero at the alpha = 0.05 level

*APC* annual percentage change, *AAPC* average annual percent change, *CI* confidence interval

## Trends in age-standardized cervical cancer incidence and death rates using joinpoint regression analysis across the states of India

Table 2 shows the Age-standardized rates and AAPC of Cervical Cancer incidence and Death in India and its states in 1990 - 2019. The age-standardized incidence and death rates of cervical cancer in India is found to be 13.10 (95% UI 10.18,17.09) and 7.38 (95% UI 5.71,10.13) per 100,000 females, respectively.

**Table 2:** Age-standardized incidence and death rates of cervical cancer for India and its states in 2019 and their Average Annual Percentage Change (AAPC) from 1990 to 2019

State	Incidence		Death	
	Rates in 2019, 95% UI (per 100 000 females)	AAPC, 95% CI (%, 1990-2019)	Rates in 2019, 95% UI (per 100 000 females)	AAPC, 95% CI (%, 1990-2019)
India	13.10 (10.18,17.09)	-0.82* (-1.39, -0.25)	7.38 (5.71,10.13)	-1.35* (-1.96, -0.75)
Andhra Pradesh	9.81 (6.34,16.96)	-1.26* (-1.42, -1.09)	5.34 (3.50,9.67)	-1.75* (-1.88, -1.61)
Arunachal Pradesh	14.01 (9.20,20.68)	-1.05* (-1.16, -0.93)	7.99 (5.52,10.95)	-1.51* (-1.63, -1.39)
Assam	10.68 (7.49,19.26)	-0.50 (-1.23, 0.24)	6.39 (4.63,12.52)	-1.19* (-1.73, -0.64)
Bihar	11.46 (7.79,18.89)	-0.49* (-0.55, -0.43)	6.55 (4.49,11.13)	-1.01* (-1.12, -0.89)
Chhattisgarh	14.81 (10.36,20.30)	-0.92* (-1.43, -0.41)	8.71 (6.30,12.97)	-1.14* (-1.64, -0.64)
Delhi	11.87 (8.50,15.78)	-2.07* (-2.51, -1.62)	7.11 (4.81,9.26)	-2.63* (-3.42, -1.82)
Goa	7.02 (4.55,11.46)	-1.31* (-1.67, -0.96)	3.58 (2.35,6.27)	-1.99* (-2.05, -1.93)
Gujarat	13.26 (9.31,18.80)	-0.53 (-1.47, 0.42)	7.12 (5.04,10.56)	-1.29* (-2.10, -0.47)
Haryana	7.85 (5.20,15.03)	-0.62 (-1.65, 0.42)	4.32 (2.85,9.54)	-1.07 (-2.21, 0.10)
Himachal Pradesh	11.66 (8.05,16.25)	-2.30* (-2.82, -1.77)	6.49 (4.70,8.81)	-2.59* (-3.33, -1.85)
Jammu & Kashmir and Ladakh	6.13 (3.95,15.71)	-0.62* (-0.76, -0.48)	3.39 (2.21,8.78)	-1.06* (-1.17, -0.94)
Jharkhand	13.18 (9.84,18.37)	-2.21* (-2.57, -1.85)	7.87 (5.84,10.94)	-2.74* (-3.06, -2.42)
Karnataka	19.83 (11.86,27.51)	-0.31 (-1.00, 0.39)	11.14 (6.67,15.10)	-0.77 (-1.61, 0.07)
Kerala	9.35 (6.57,12.92)	-1.07 (-2.21, 0.08)	4.77 (3.29,6.49)	-1.88* (-2.41, -1.34)
Madhya Pradesh	13.41 (9.77,17.92)	-0.64* (-0.85, -0.44)	7.88 (5.85,10.47)	-1.05* (-1.25, -0.86)
Maharashtra	16.75 (11.08,22.65)	-0.41 (-0.94, 0.13)	9.26 (6.22,12.29)	-1.04* (-1.59, -0.49)
Manipur	9.25 (5.90,14.93)	-0.87* (-1.13, -0.61)	5.59 (3.78,8.86)	-1.10* (-1.68, -0.51)
Meghalaya	11.40 (7.59,16.99)	-1.14* (-1.56, -0.71)	6.84 (4.86,9.84)	-1.42* (-1.61, -1.24)
Mizoram	18.99 (9.50,29.21)	-0.72* (-1.26, -0.18)	10.90 (5.64,16.15)	-1.11* (-1.72, -0.50)
Nagaland	12.95 (7.59,19.42)	-1.23* (-1.36, -1.10)	7.57 (4.81,10.93)	-1.55* (-1.67, -1.42)
Odisha	7.10 (4.42,17.34)	-1.32* (-1.96, -0.67)	4.22 (2.64,10.63)	-1.66* (-1.88, -1.44)
Other Union Territories	9.50 (6.34,14.04)	-1.44* (-1.74, -1.14)	5.14 (3.56,7.72)	-1.85* (-2.01, -1.68)
Punjab	13.00 (9.65,17.82)	-0.43* (-0.83, -0.02)	7.14 (5.10,9.72)	-0.95* (-1.46, -0.44)
Rajasthan	11.77 (8.32,17.92)	0.05 (-0.71, 0.82)	6.26 (4.25,10.45)	-0.65 (-1.53, 0.25)
Sikkim	9.72 (6.61,14.78)	-1.35* (-1.78, -0.93)	5.47 (3.90,7.88)	-1.85* (-2.05, -1.65)
Tamil Nadu	19.91 (11.27,26.83)	-1.49* (-1.94, -1.04)	11.57 (6.31,15.59)	-1.98* (-2.29, -1.67)
Telangana	9.76 (6.03,16.26)	-1.47* (-1.52, -1.41)	5.07 (3.23,9.03)	-2.08* (-2.18, -1.98)
Tripura	10.64 (7.00,15.99)	-1.36* (-1.51, -1.21)	6.25 (4.36,9.15)	-1.70* (-1.82, -1.57)
Uttar Pradesh	13.48 (9.21,21.21)	-0.26 (-0.74, 0.22)	7.79 (5.43,13.89)	-0.72* (-1.15, -0.30)
Uttarakhand	12.32 (8.80,19.28)	-1.22* (-1.33, -1.11)	6.83 (4.94,11.45)	-1.74* (-1.83, -1.66)
West Bengal	9.67 (7.06,13.94)	-1.11* (-1.87, -0.35)	5.24 (3.89,7.89)	-1.83* (-3.02, -0.62)

For Incidence, all of the states have shown a significant declining trend except Gujarat (AAPC: −0.53; 95%CI: −1.47 to 0.42; *p* > 0.05), Assam (AAPC: −0.50; 95%CI: −1.23 to 0.24; *p* > 0.05) , Haryana (AAPC: −0.62; 95%CI: −1.65 to 0.42; *p* > 0.05), Karnataka (AAPC: −0.31; 95%CI: −1.00 to 0.39; *p* > 0.05) , Maharashtra (AAPC: −0.41; 95%CI: -0.94 to 0.13; *p* > 0.05) , Uttar Pradesh (AAPC: −0.26; 95%CI: −0.74 to 0.22; *p* > 0.05) and Kerala (AAPC: −1.07; 95%CI: −2.21 to 0.08; *p* > 0.05) where non-significant declining trend was observed and Rajasthan (AAPC: 0.05; 95%CI: −0.71 to 0.82; *p* > 0.05) where non-significant increasing trend is observed (Table 2, Supplementary Fig. 1). All of the states have shown a significant declining trend in mortality except Haryana (AAPC: −1.07; 95%CI: −2.21 to 0.10; *p* > 0.05), Karnataka (AAPC: −0.77; 95%CI: −1.61 to 0.07; *p* > 0.05) and Rajasthan (AAPC: −0.65; 95%CI: −1.53 to 0.25; *p* > 0.05), where non-significant declining trend is observed (Table 2, Supplementary Fig. 2).

## Discussion

In this study, we found that there has been a significant decline in the incidence and mortality of cervical cancer over the past three decades in India; This corroborates with studies conducted by various others [16–19]. Whereas in high-income countries, cervical cancer incidence and mortality have decreased by more than half over the past 30 years after the introduction of formalized screening programmes [20]. Recent evidence suggests that factors such as socioeconomic development and high-income countries type lifestyle-related transitions underpin changes in cancer risk, reducing the cervical cancer rates in countries with emerging economies [1, 21]. A trend of decline in the incidence rates in urban areas truly represents the societal changes that are not reflected in India's rural areas [22].

Based on complete and reliable data obtained from the Bombay Cancer Registry, a paper emphasizes that the decline in the incidence of cervical is not due to change in registration practice but solely attributable to epidemiological transition [23, 24]. In lower-middle-income countries, a decline in cervical cancer incidence has been due to opportunistic screening [25]. Bobdey and colleagues have found that data from most major Indian cancer registries indicated a decreasing trend of cervical cancer; however, the decrease was small [19]. Further, research has also highlighted that the decline in the incidence of cervical cancer is also because of the lack of an organized mass screening program for the early detection of cervical cancer in India [26].

Notwithstanding with the caveat of the estimates from various information, the absolute number of cases of cervix uteri cancer has increased over time (GLOBCAN) (471000 in 2000, 529000 in 2008, 570,000 in 2018) [27, 28]. The rising age at marriage, increase in the age at first term pregnancy, lowering parity could have contributed to reducing the risk of HPV acquisition, decreasing the incidence of cervical cancer in India. Further, vaccination could have led to this significant decline in the burden of cervical cancer. As evident from developed countries, screening and vaccination have been identified as preventive measures in reducing cervical cancer burden [29]. Consistent with our findings, a study conducted across 38 countries found a substantial decrease in the age-standardized incidence rates in the highest-income countries. However, these rates were found to increase, or stabilized rates were visible in lower-resourced settings [24].

This study reports a substantial decline in the mortality due to cervical cancer in the country. This finding is consistent with other studies as well. The decline in the morality is mainly attributable to improved health facilities, screening and vaccination coverage. Arbyn reported that the proportion of deaths due to cervical cancer has decreased from 8.2% in 2008 to 7.5% in 2018 [30]. Chauhan and colleagues found that introducing HPV vaccination alone led to a 60% decline in cervical cancer-related mortality compared to those without any vaccination and screening [31]. This study reveals that different screening strategies have a varying reduction in lifetime occurrence of cervical cancer caused by HPV from 16 to 61% and reducing mortality due to cervical cancer from 28 to 70% [31]. Furthermore, a successful organized and opportunistic screening has led to a substantial decline in the last 50 years in cervical cancer morbidity and mortality in high- and middle-income countries [32].

There has been a -21.32 percentage change in the incidence of cervical cancer in the period 1990-2019. Similarly, a change of -32.29 percentage point is observed in the country's mortality due to cervical cancer. The trends observed in cervical cancer incidence and mortality in the country over the period are likely due to population ageing, changes in knowledge and literacy, early screening, improved access to health care, and other risk factors. Studies have also highlighted the lack of inadequate and incomplete information on deaths leading to the inaccuracy of mortality statistics and trends. Reproductive risk factors such as later age at first birth, lower parity is inversely related to decreasing age-standardized incidence rates [33]. The age-standardized incidence and death rates of cervical cancer are 13.10 and 7.38 per 100,000 females. Consistent with our findings, researchers found the age-standardized incidence and mortality rates of cervical cancer to be 14.7 and 9.2 per 100,000 among Indian women [34].

Similarly, researchers at George institute also found the age-standardized incidence and mortality rates of cervical cancer to be 22 and 12.4 per 100,000 women. The rank of age-standardized cervical cancer incidence rate has decreased significantly in states like Arunachal Pradesh and Assam. A geographical difference reflected in the incidence of mortality is due to differences in exposure to risk factors and serious inequalities in access to adequate screening and effective cancer treatment facilities.

Even after more than a decade of the introduction of HPV vaccines, the prevalence of cervical cancer is quite alarming. In India, Universal cervical cancer screening is an unmet need [35]. The fourth round of the National Family Health Survey estimates that only 22.3% of eligible women received cervical cancer screening during 2015-16 [36]. The associated mortality with Cervical cancer in India is one of the highest in the world [37–39]. Studies have reported that India's overall knowledge and awareness about cervical cancer, HPV, and HPV vaccination is very poor. Researchers are of the opinion that factors such as societal, religious and prejudiced ideas, socioeconomic status, including lack of knowledge, awareness and attitude, affect the HPV vaccination in India. The present study has a few limitations. First, Although the GBD study has subsumes various methods to improve the quality of data by adjusting for missing or incomplete data, but we can't rule out the possibility of some inaccuracy in the mortality data. Second, this is an ecological study; hence, interpretations from this study are true at population levels, but they do not necessarily hold at the individual level.

## Conclusion

This study concludes that the overall incidence and mortality of cervical cancer showed a significant decreasing trend in India between 1990 and 2019, the highest decline in the incidence and mortality rates were reported in the period 1998-2005. The highest incidence and mortality of cervical cancer were reported in Tamilnadu and lowest in Jammu & Kashmir and Ladakh during 1990 & 2019. The highest percentage decrement in the incidence of cervical cancer was reported in Jharkhand and lowest in Jammu & Kashmir during the study period. The highest percentage decrement in cervical cancer mortality was seen in Jharkhand from 1990 to 2019.

Though the incidence and mortality of cervical cancer declined over the past three decades but it is still a major public health problem in India. Information, education and communication activities for girls, boys, parents and the community regarding the risk factors of cervical cancer, mode of transmission, screening programme, HPV, HPV vaccination and treatment modalities should be provided throughout the country. HPV vaccine should be included in the national immunization program to improve its availability and accessibility to all eligible beneficiaries. The cervical cancer screening facility should be available at a peripheral level for early diagnosis of precancerous conditions. The involvement of non-government organizations can play a key role in primary, secondary and tertiary levels of prevention for cervical cancer.

## Supplementary Information

**Additional file 1: Supplementary Table 1.** Percentage changes in cervical cancer incidence among women of all ages in India and its states over the period 1990 to 2019. **Supplementary Table 2.** Percentage changes in cervical cancer mortality among women of all ages in India and its states over the period 1990 to 2019. **Supplementary Figure 1.** Trends in age standardized cervical cancer Incidence rate using joinpoint regression analysis across states of India. **Supplementary Figure 2.** Trends in age standardized mortality rate of cervical cancer using joinpoint regression analysis across states of India

## Data Availability

Data was extracted from an online tool produced by the IHME, which is publicly available called the GHDx (Global Health Data Exchange) query tool (http://ghdx.healthdata.org/gbd-results-tool)
