# Supplementary material for: Secular trends in incidence and mortality of cervical cancer in India and its states, 1990-2019: data from the Global Burden of Disease 2019 Study
Source: BMC Cancer. 2022 Feb 7;22:149. doi: 10.1186/s12885-022-09232-w (PMC8819855; doi:10.1186/s12885-022-09232-w)
Supplement: Supplementary file 1 — Additional file 1: Supplementary Table 1. Percentage changes in cervical cancer incidence among women of all ages in India and its states over the period 1990 to 2019. Supplementary Table 2. Percentage changes in cervical cancer mortality among women of all ages in India and its states over the period 1990 to 2019. Supplementary Figure 1. Trends in age standardized cervical cancer Incidence rate using joinpoint regression analysis across states of India. Supplementary Figure 2. Trends in age standardized mortality rate of cervical cancer using joinpoint regression analysis across states of India [file 12885_2022_9232_MOESM1_ESM.docx]

Supplementary Table 1: Percentage changes in cervical cancer incidence among women of all ages in India and its states over the period 1990 to 2019.

| State | Percentage Changes in Cervical Cancer Incidence | | | |
| --- | --- | --- | --- | --- |
|  | **1990-2000** | **2000-2010** | **2010-2019** | **1990-2019** |
| Andhra Pradesh | -11.45 | -14.79 | -8.21 | -30.74 |
| Arunachal Pradesh | -16.54 | -4.13 | -7.47 | -25.96 |
| Assam | 5.98 | -16.37 | -6.59 | -17.22 |
| Bihar | -6.86 | -1.81 | -5.68 | -13.73 |
| Chhattisgarh | -11.65 | -4.09 | -7.69 | -21.78 |
| Delhi | -14.99 | -26.98 | -12.40 | -45.62 |
| Goa | -13.91 | -10.33 | -10.59 | -30.98 |
| Gujarat | -23.27 | -3.08 | 14.07 | -15.17 |
| Haryana | -4.80 | -15.85 | 4.23 | -16.49 |
| Himachal Pradesh | -19.72 | -30.87 | -6.92 | -48.34 |
| Jammu & Kashmir and Ladakh | -12.83 | -1.59 | -1.27 | -15.31 |
| Jharkhand | -28.47 | -16.75 | -16.40 | -50.22 |
| Karnataka | -8.96 | -17.48 | 22.11 | -8.26 |
| Kerala | -2.85 | -19.44 | -6.35 | -26.71 |
| Madhya Pradesh | -6.01 | -12.94 | -0.21 | -18.34 |
| Maharashtra | -6.31 | -23.86 | 22.16 | -12.85 |
| Manipur | -13.18 | -3.15 | -6.58 | -21.45 |
| Meghalaya | -15.50 | -10.06 | -6.39 | -28.85 |
| Mizoram | -10.54 | -0.93 | -10.24 | -20.45 |
| Nagaland | -15.88 | -10.64 | -6.39 | -29.63 |
| Odisha | -5.16 | -15.98 | -13.84 | -31.35 |
| Other Union Territories | -14.44 | -15.43 | -9.48 | -34.50 |
| Punjab | -1.57 | -19.68 | 11.45 | -11.89 |
| Rajasthan | -1.23 | -21.83 | 30.02 | 0.38 |
| Sikkim | -15.15 | -5.46 | -14.84 | -31.69 |
| Tamil Nadu | -8.59 | -23.64 | -7.76 | -35.61 |
| Telangana | -15.24 | -12.10 | -11.21 | -33.85 |
| Tripura | -15.22 | -11.28 | -8.41 | -31.10 |
| Uttar Pradesh | -9.76 | -17.36 | 23.62 | -7.82 |
| Uttarakhand | -7.50 | -11.54 | -14.80 | -30.28 |
| West Bengal | 2.67 | -28.57 | -3.29 | -29.08 |
| India | -8.86 | -17.88 | 5.12 | -21.32 |

Supplementary Table 2: Percentage changes in cervical cancer mortality among women of all ages in India and its states over the period 1990 to 2019.

| State | Percentage Changes in Cervical Cancer Mortality | | | |
| --- | --- | --- | --- | --- |
|  | **1990-2000** | **2000-2010** | **2010-2019** | **1990-2019** |
| Andhra Pradesh | -14.30 | -21.35 | -11.59 | -40.41 |
| Arunachal Pradesh | -18.56 | -11.04 | -10.52 | -35.18 |
| Assam | 4.15 | -18.01 | -13.34 | -26.01 |
| Bihar | -8.66 | -10.14 | -8.38 | -24.80 |
| Chhattisgarh | -12.74 | -8.42 | -8.96 | -27.25 |
| Delhi | -15.98 | -30.62 | -16.51 | -51.33 |
| Goa | -16.05 | -19.90 | -14.74 | -42.67 |
| Gujarat | -27.00 | -11.98 | 7.29 | -31.06 |
| Haryana | -7.93 | -19.46 | -0.80 | -26.44 |
| Himachal Pradesh | -21.63 | -33.02 | -11.18 | -53.37 |
| Jammu & Kashmir and Ladakh | -14.49 | -9.08 | -5.15 | -26.26 |
| Jharkhand | -30.42 | -22.99 | -18.19 | -56.16 |
| Karnataka | -12.76 | -19.24 | 15.00 | -18.98 |
| Kerala | -8.41 | -28.02 | -12.06 | -42.03 |
| Madhya Pradesh | -8.25 | -16.83 | -4.93 | -27.45 |
| Maharashtra | -9.81 | -29.26 | 14.83 | -26.73 |
| Manipur | -13.52 | -8.27 | -8.62 | -27.51 |
| Meghalaya | -17.02 | -14.42 | -8.00 | -34.66 |
| Mizoram | -12.76 | -6.23 | -11.31 | -27.45 |
| Nagaland | -17.09 | -16.27 | -7.86 | -36.03 |
| Odisha | -5.80 | -21.20 | -17.01 | -38.40 |
| Other Union Territories | -14.84 | -21.76 | -12.18 | -41.49 |
| Punjab | -4.66 | -24.60 | 6.33 | -23.56 |
| Rajasthan | -5.92 | -28.62 | 23.10 | -17.34 |
| Sikkim | -16.71 | -12.61 | -18.14 | -40.41 |
| Tamil Nadu | -10.17 | -27.35 | -14.50 | -44.20 |
| Telangana | -16.69 | -20.08 | -17.21 | -44.88 |
| Tripura | -17.77 | -15.59 | -10.17 | -37.65 |
| Uttar Pradesh | -12.19 | -22.45 | 18.14 | -19.56 |
| Uttarakhand | -8.46 | -18.27 | -18.69 | -39.16 |
| West Bengal | -1.94 | -33.83 | -10.57 | -41.97 |
| India | -11.56 | -23.05 | -0.48 | -32.27 |

**Supplementary Figure 1: Trends in age standardized cervical cancer Incidence rate using joinpoint regression analysis across states of India**

| 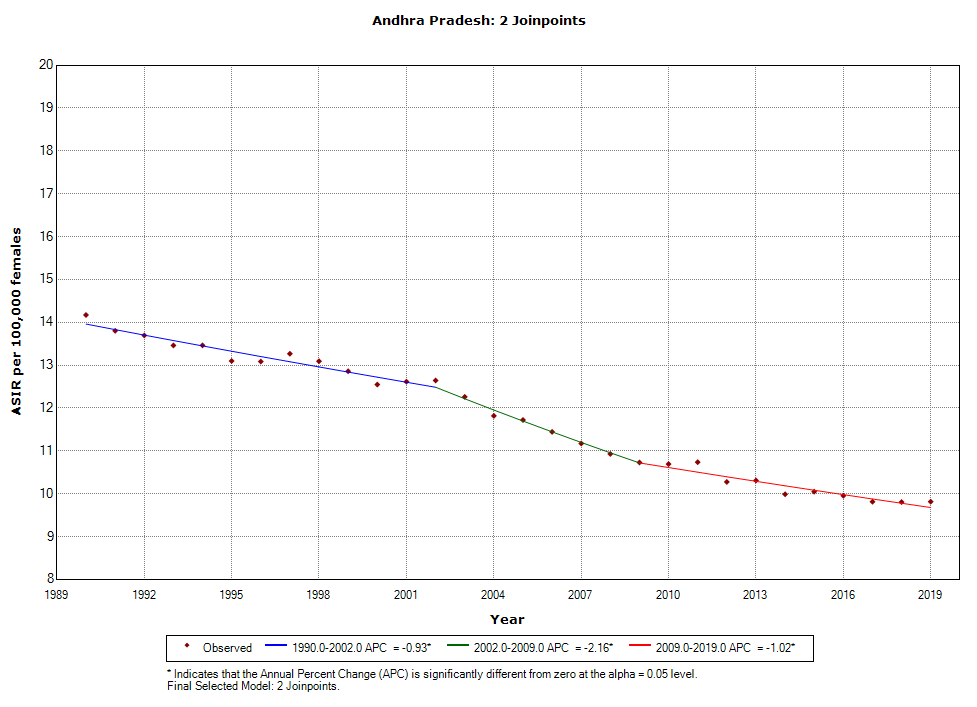 | 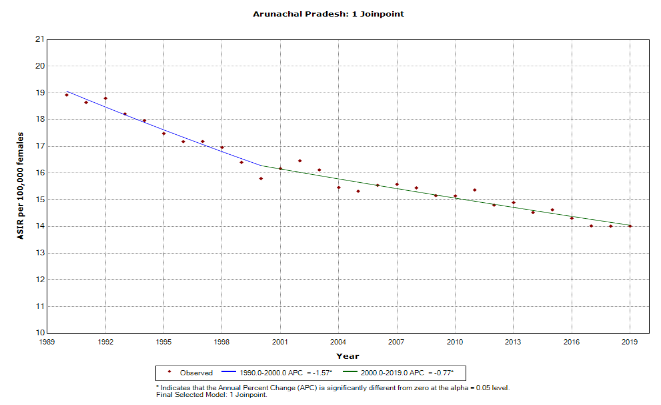 | 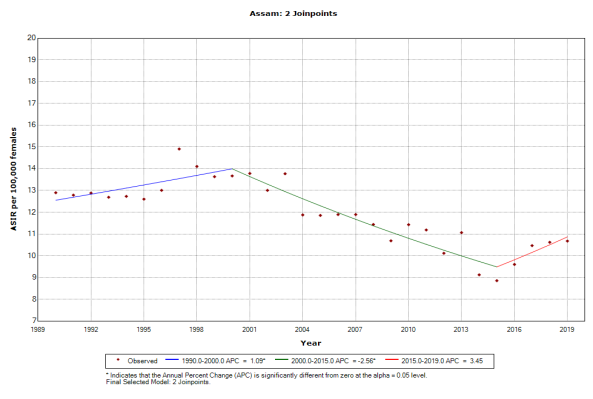 |
| --- | --- | --- |
| 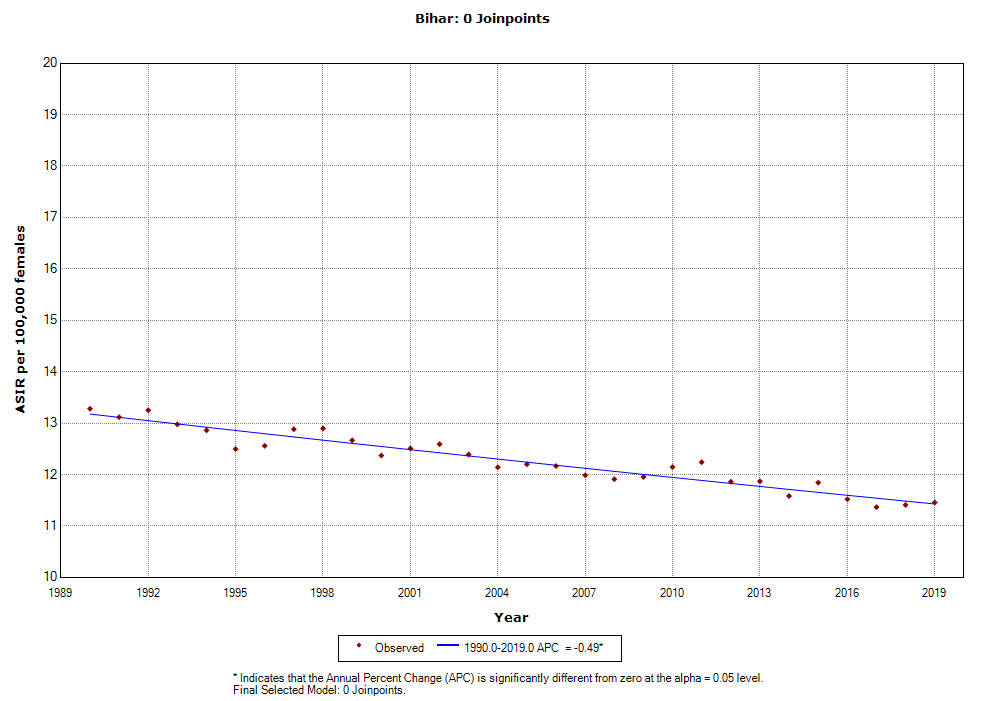 | 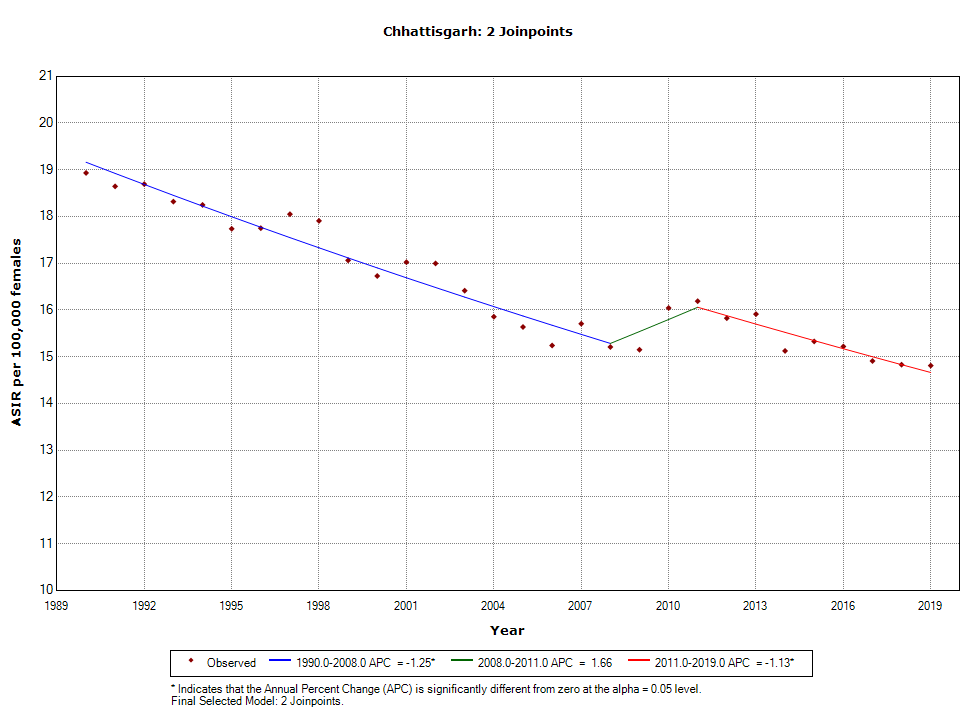 | 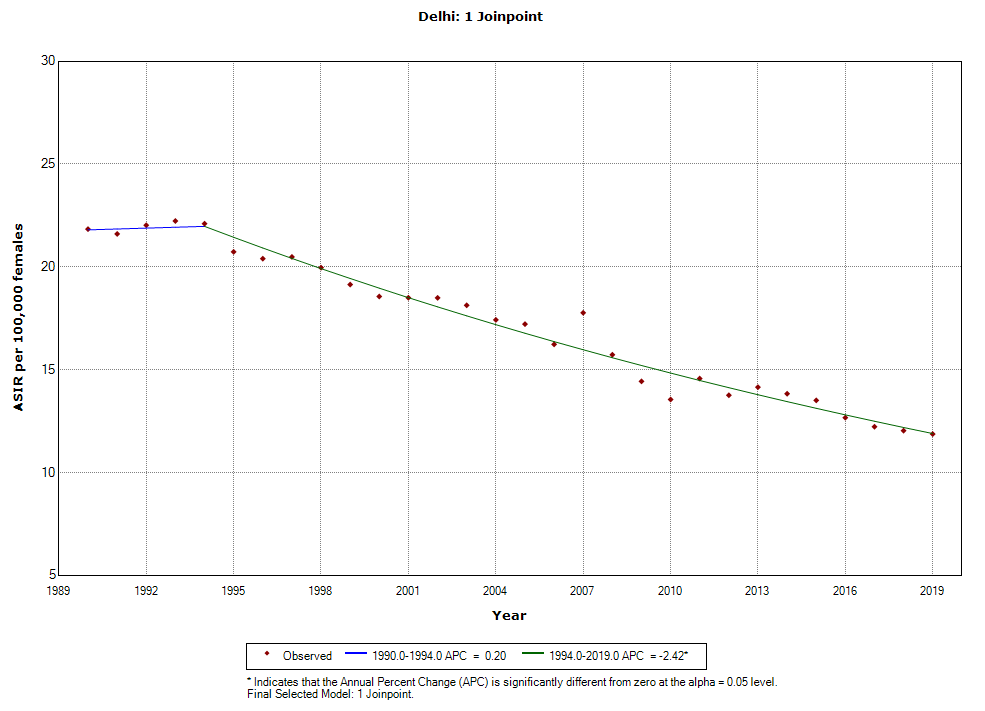 |
| 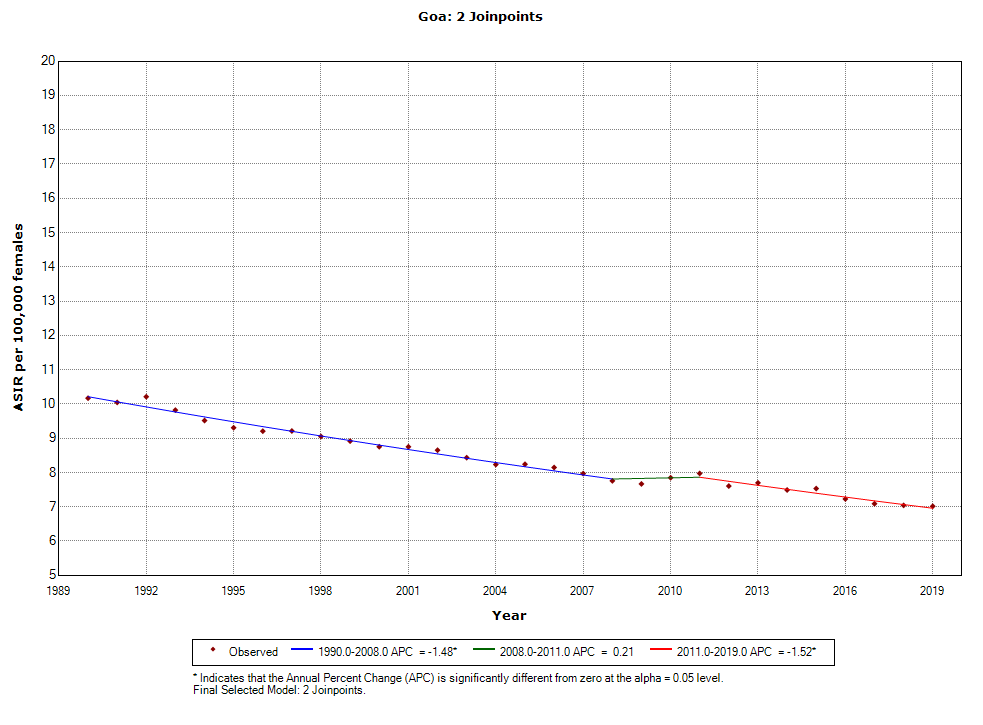 | 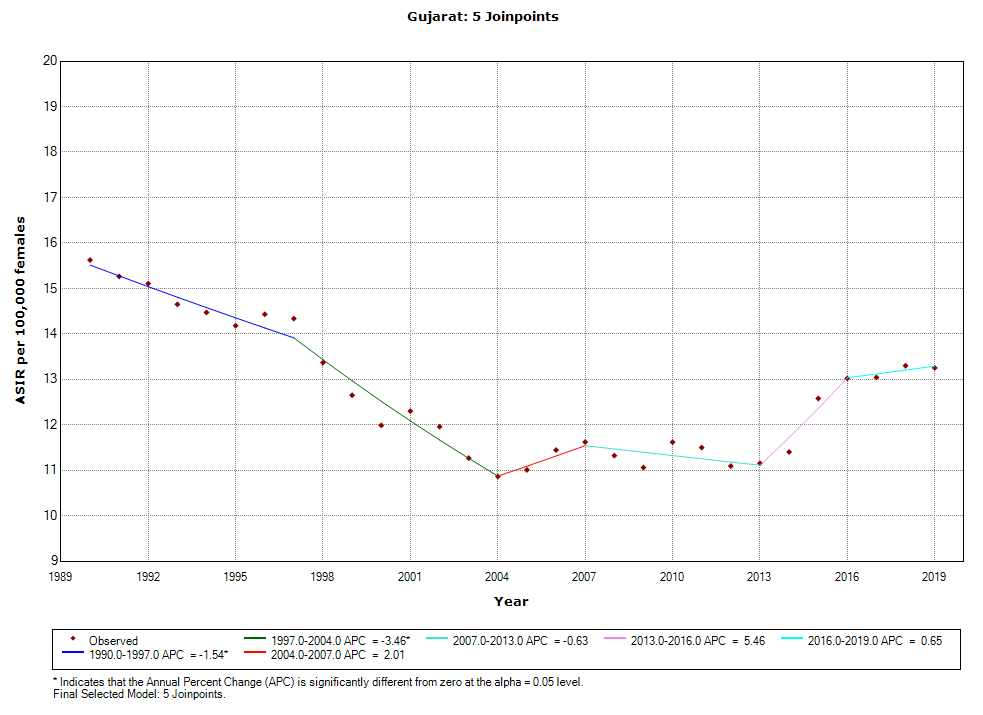 | 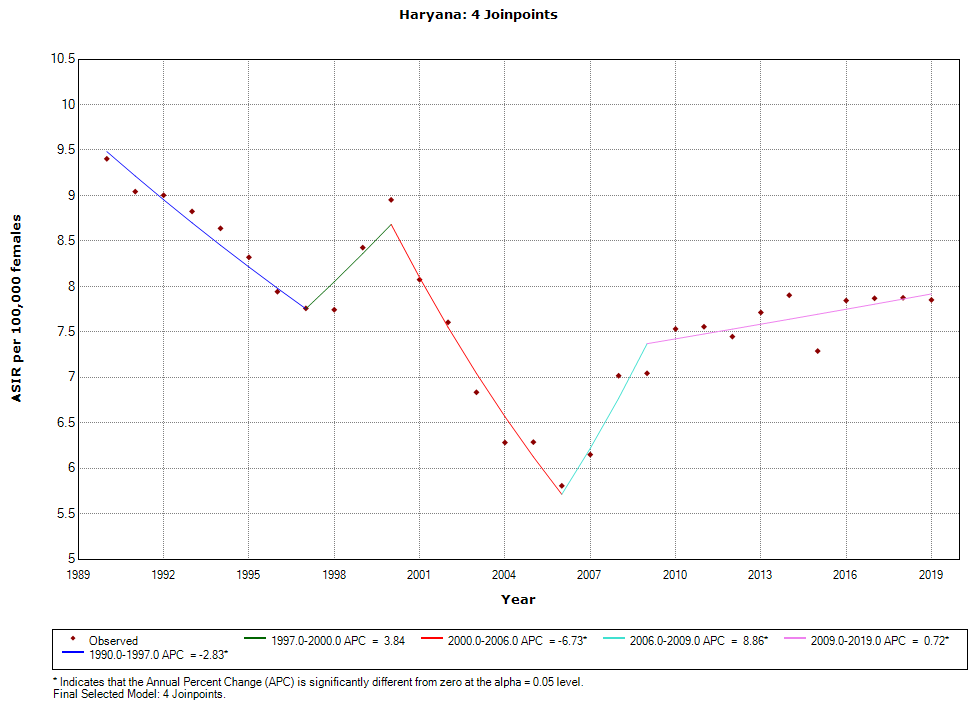 |
| 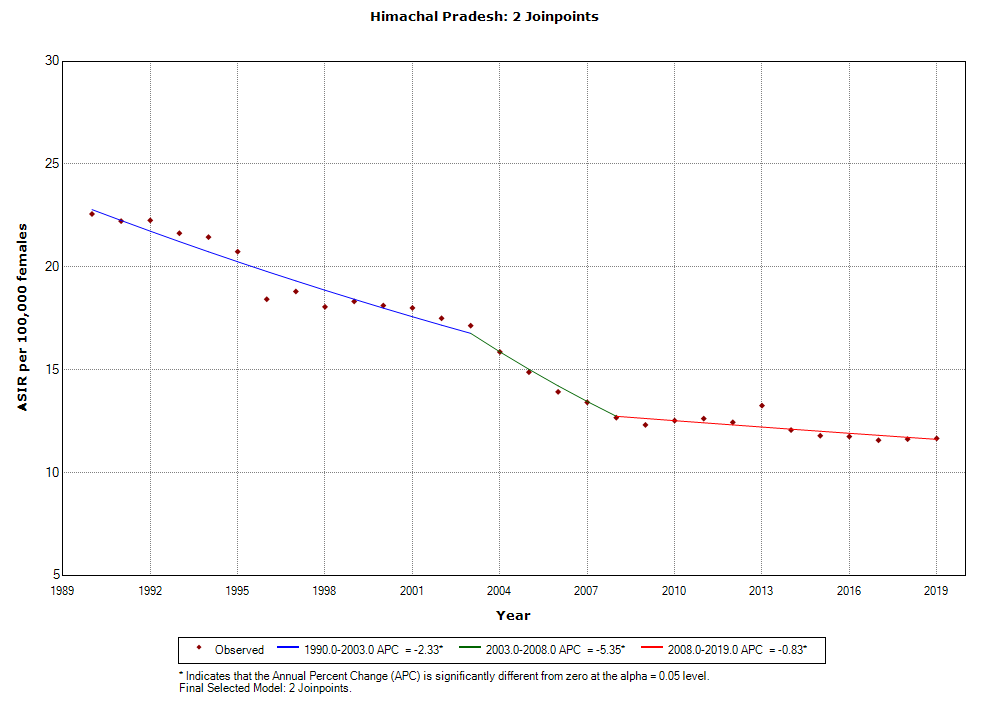 | 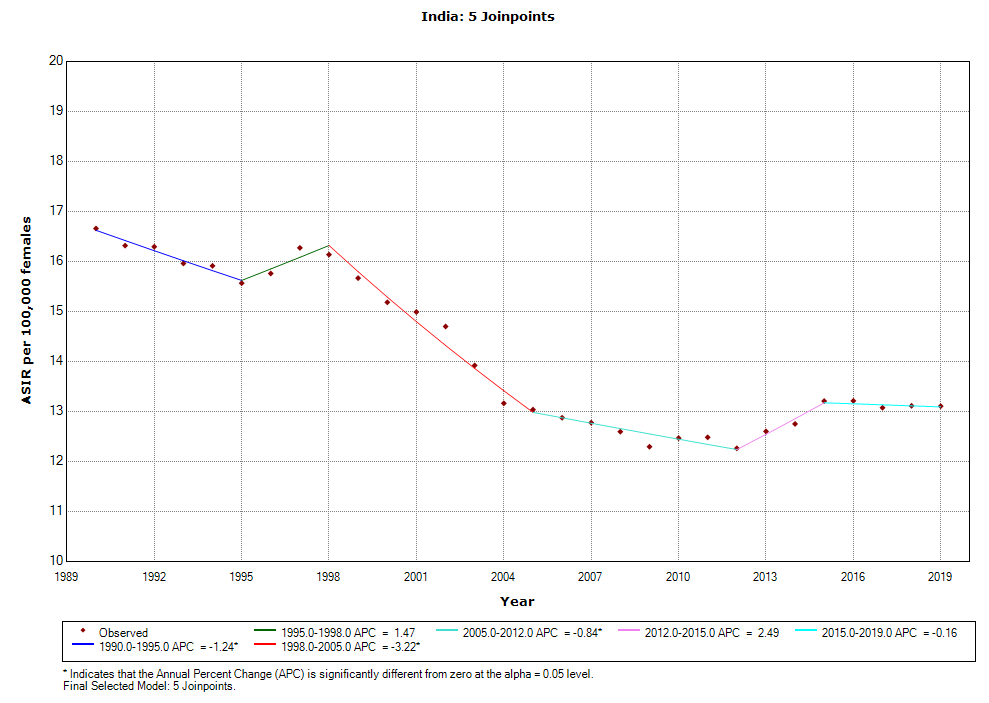 | 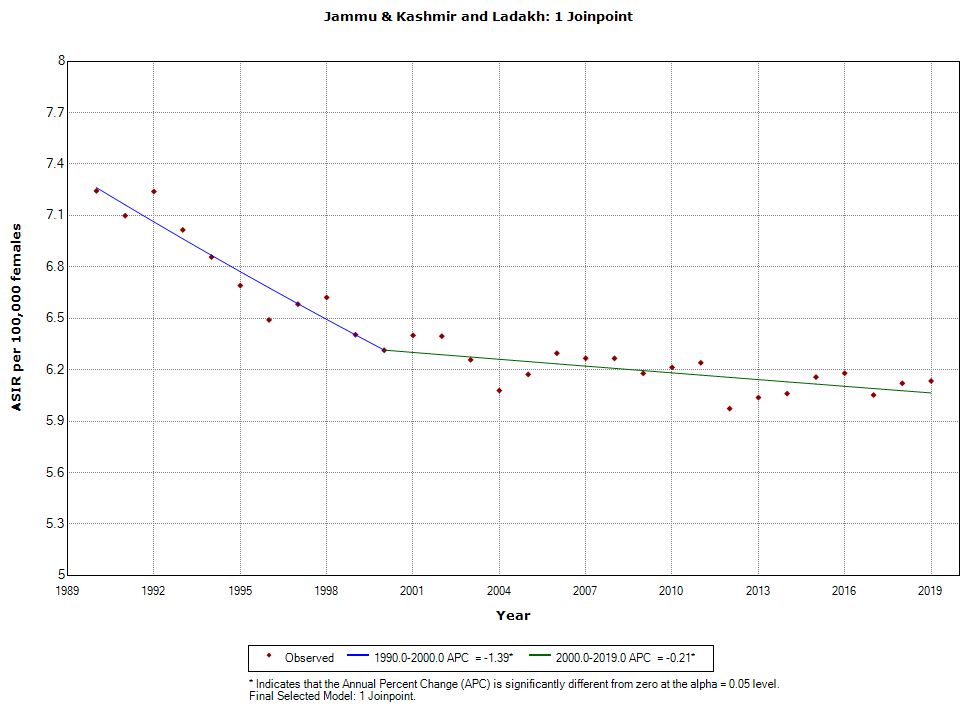 |
| 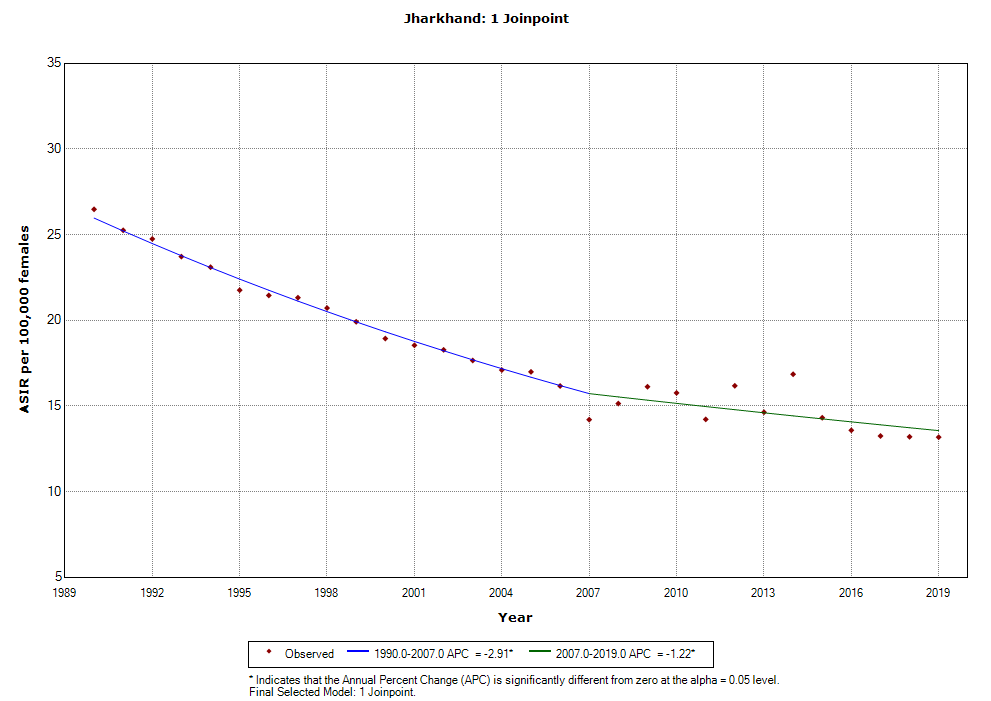 | 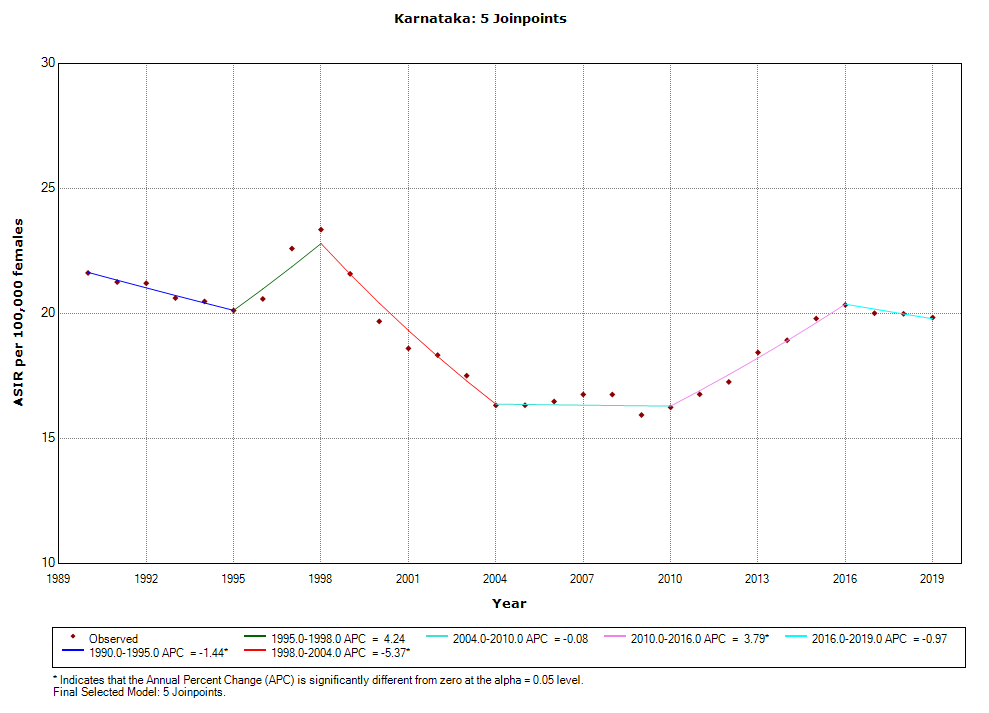 | 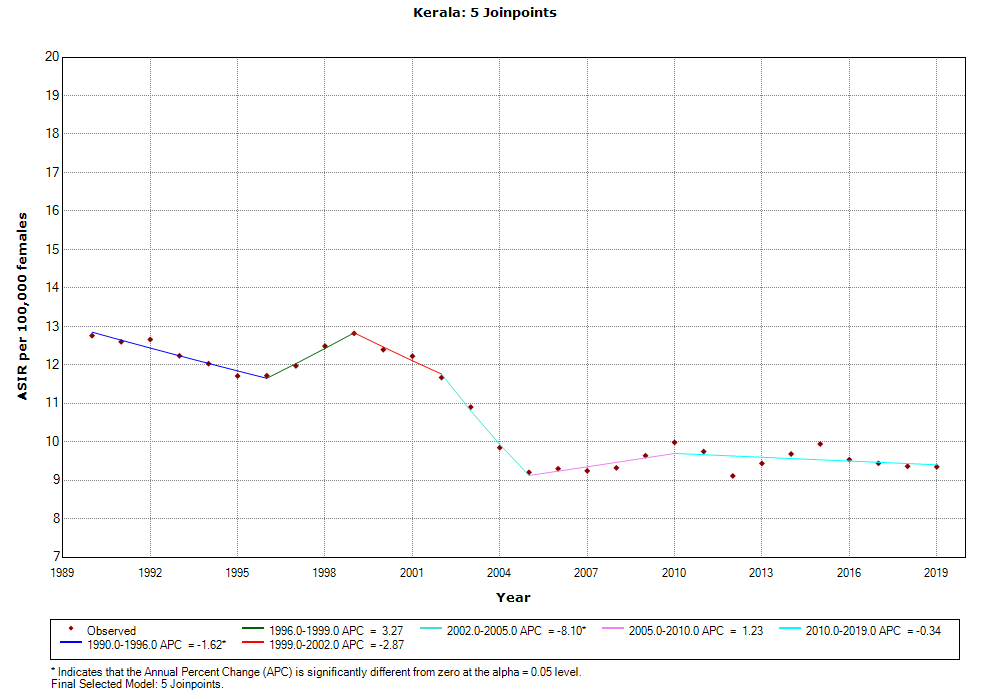 |
| 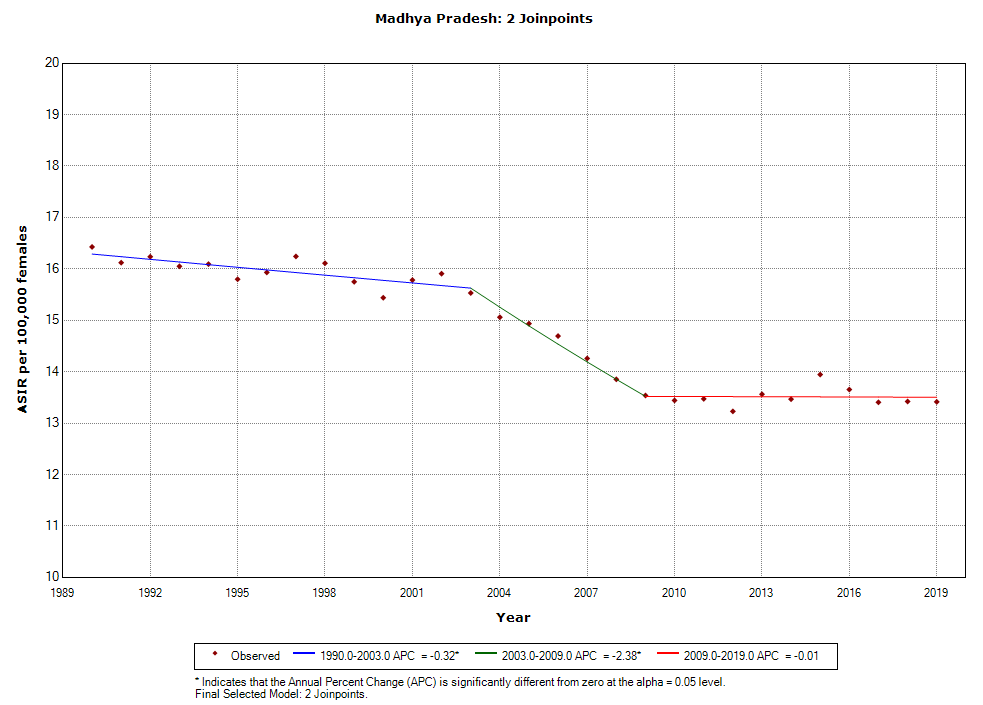 | 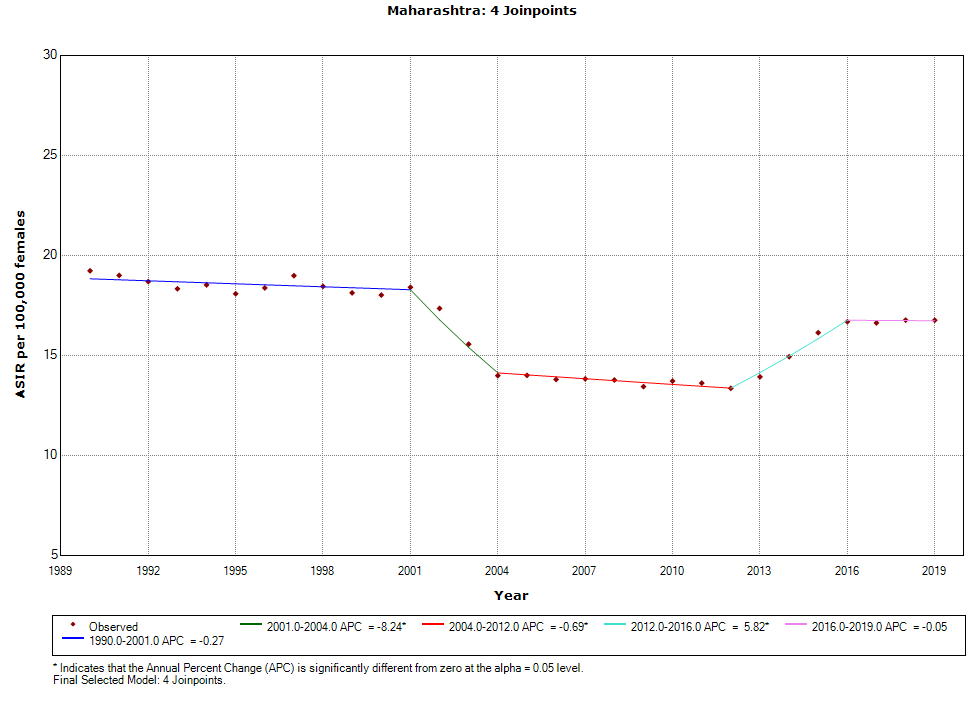 | 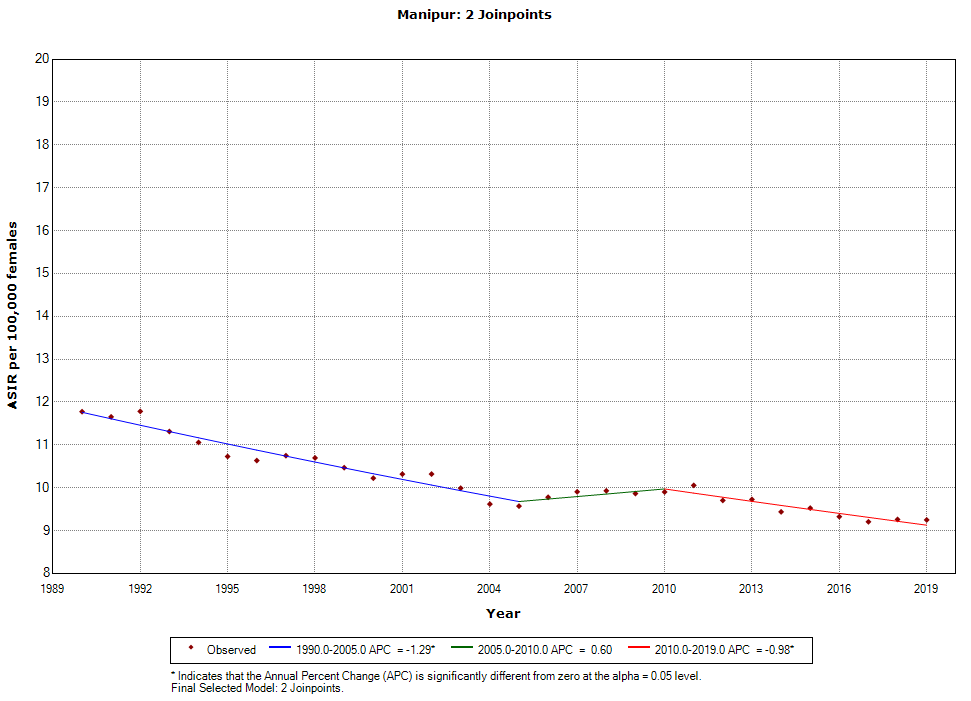 |
| 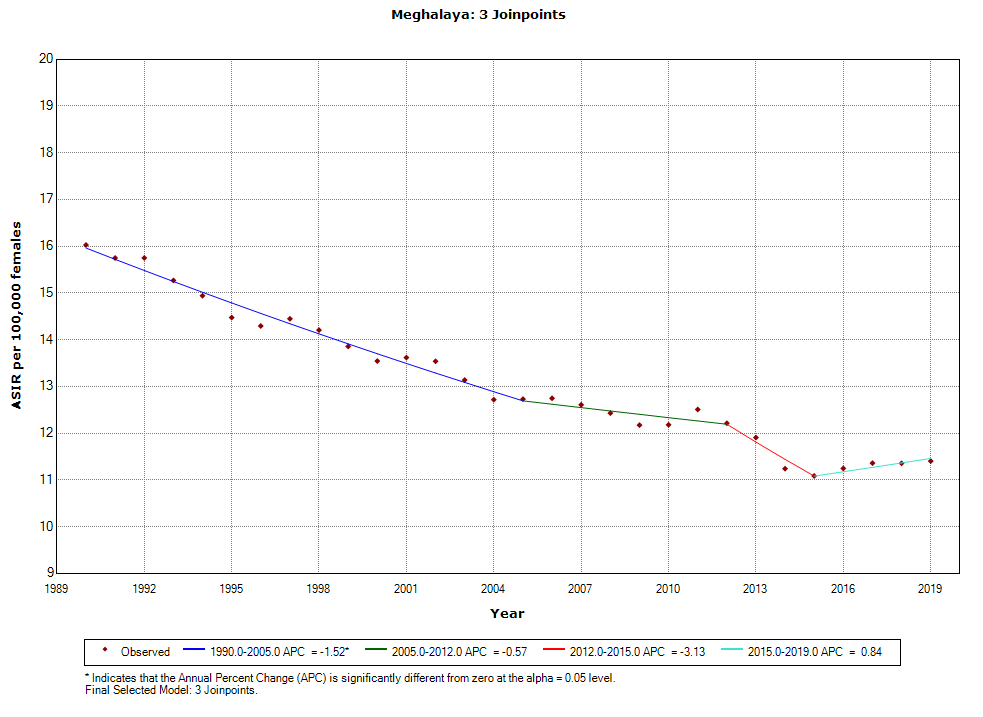 | 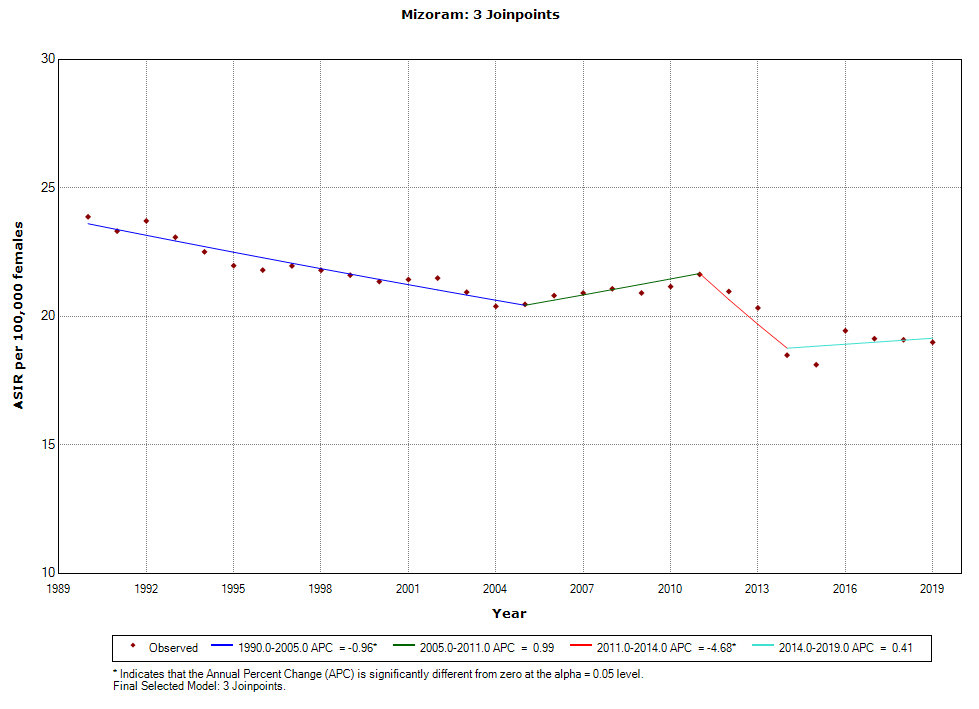 | 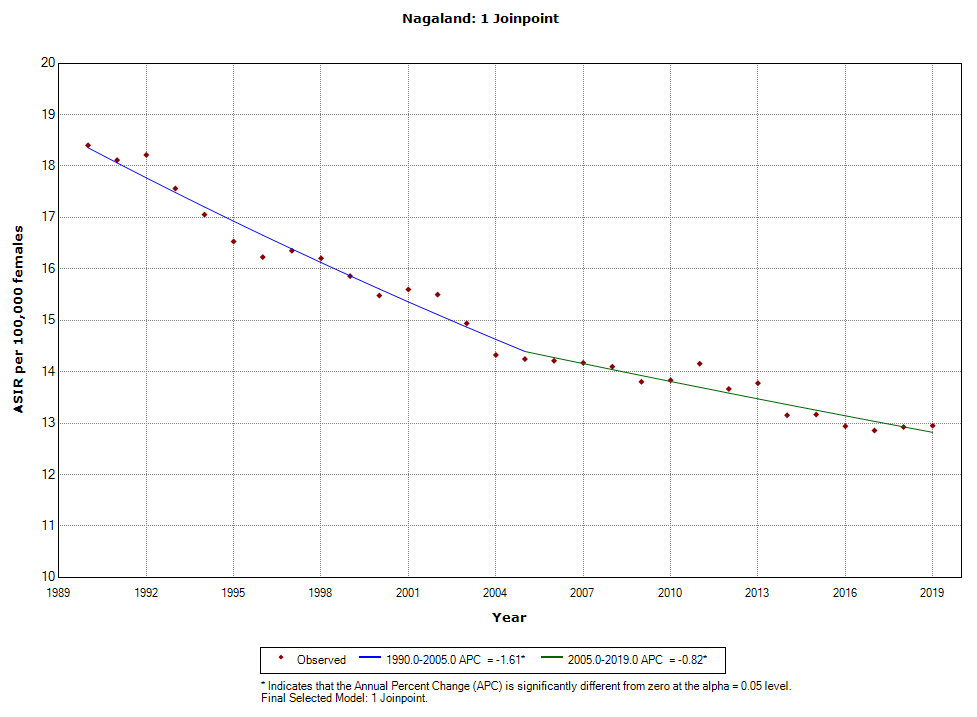 |
| 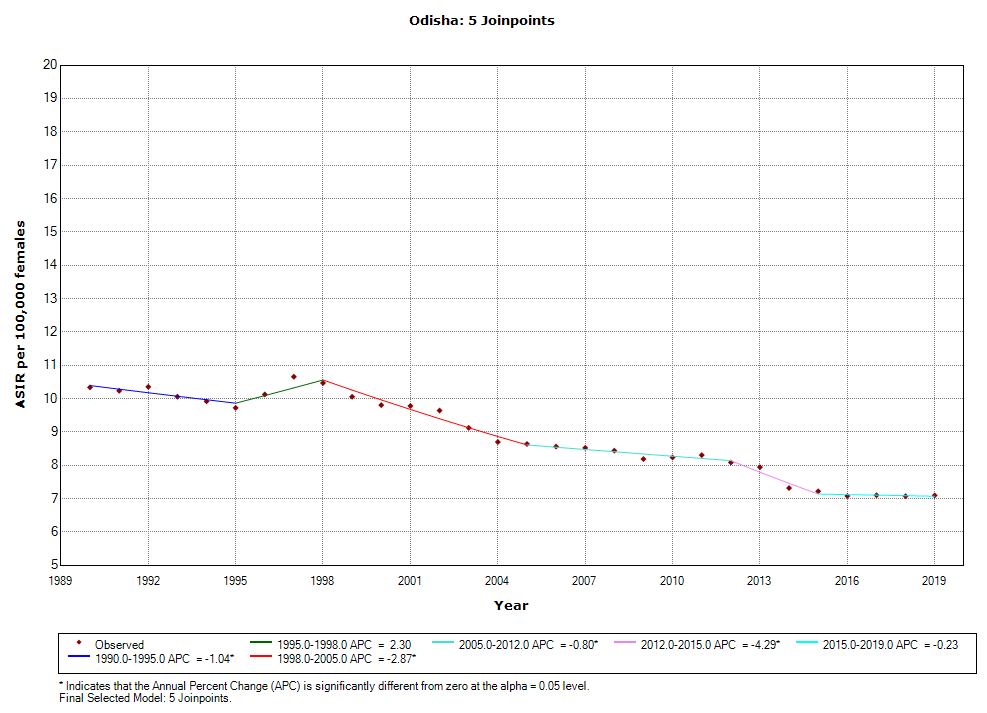 | 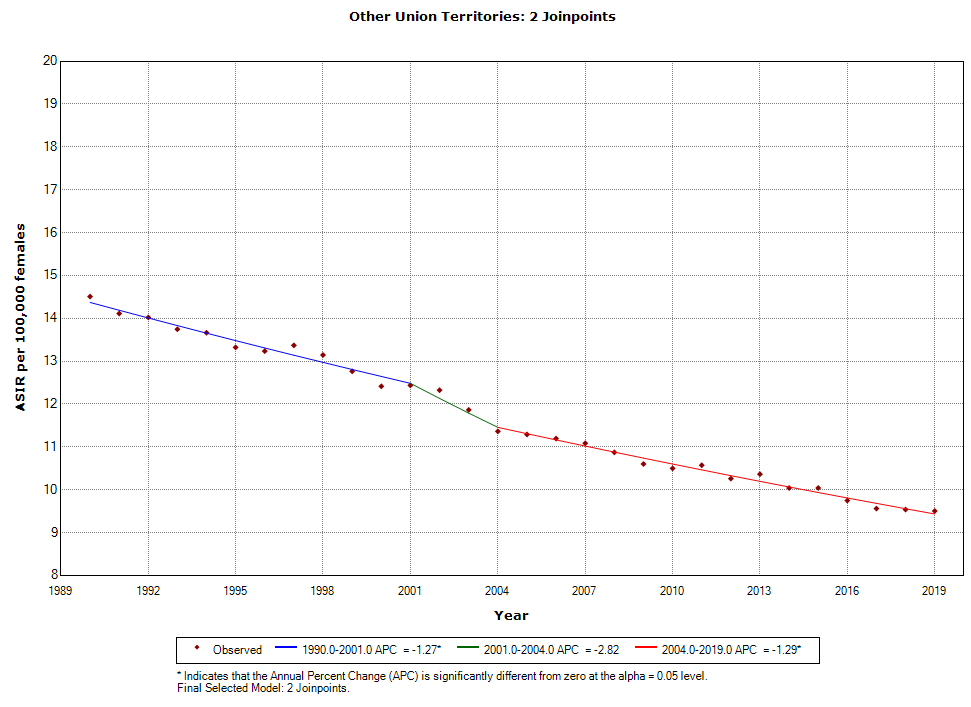 | 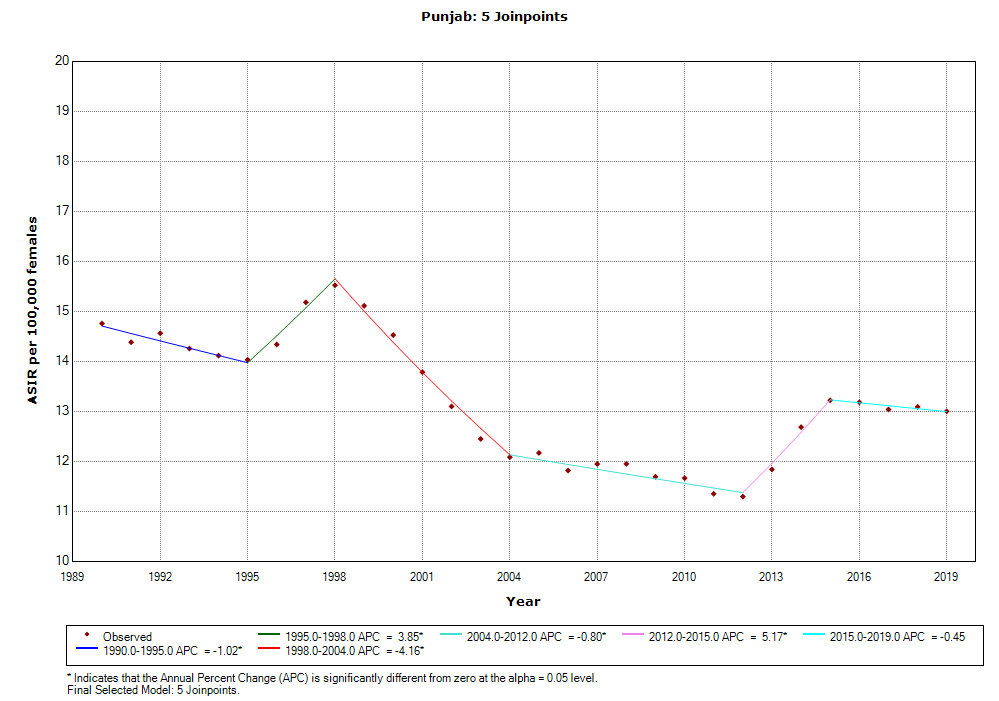 |
| 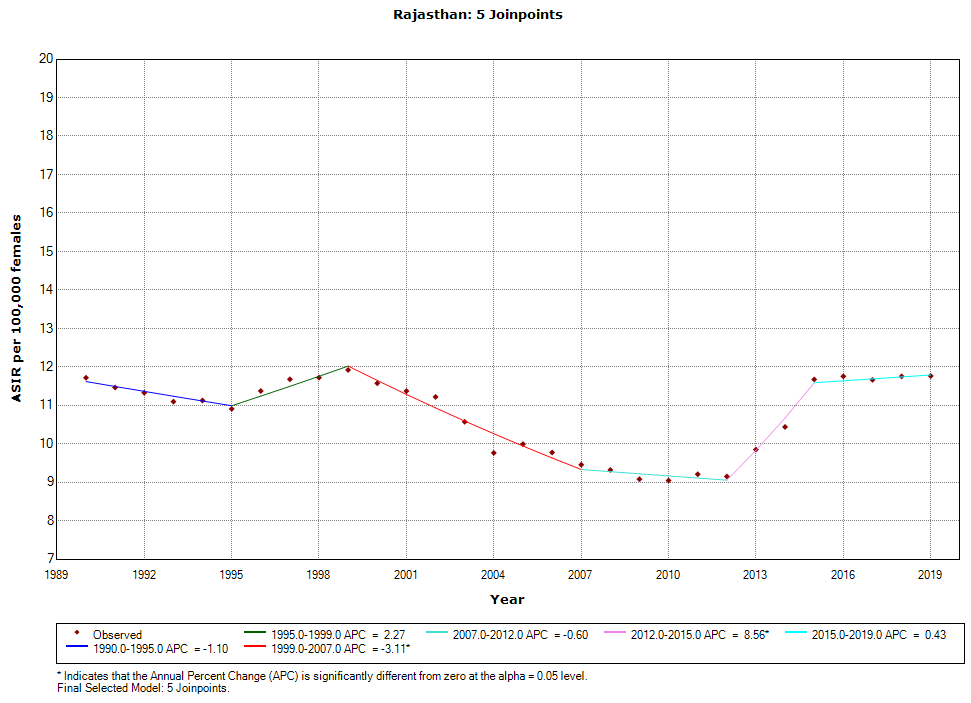 | 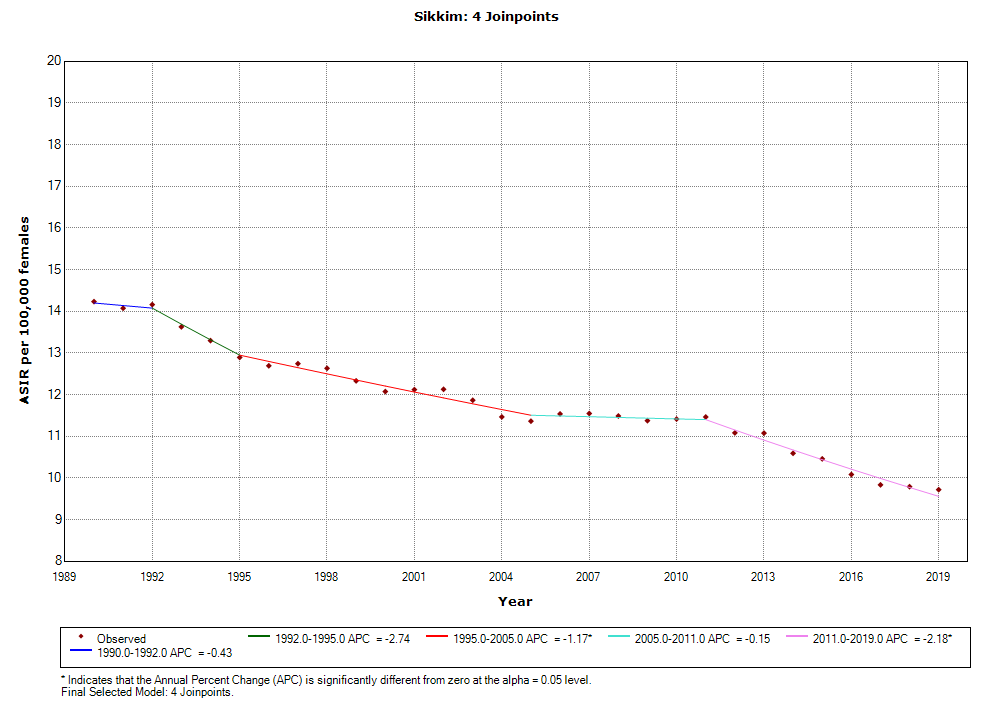 | 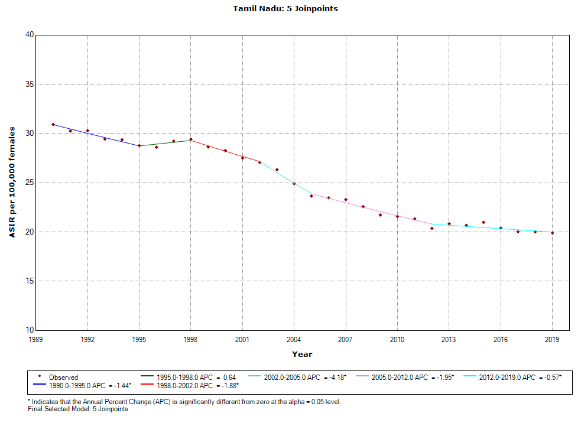 |
| 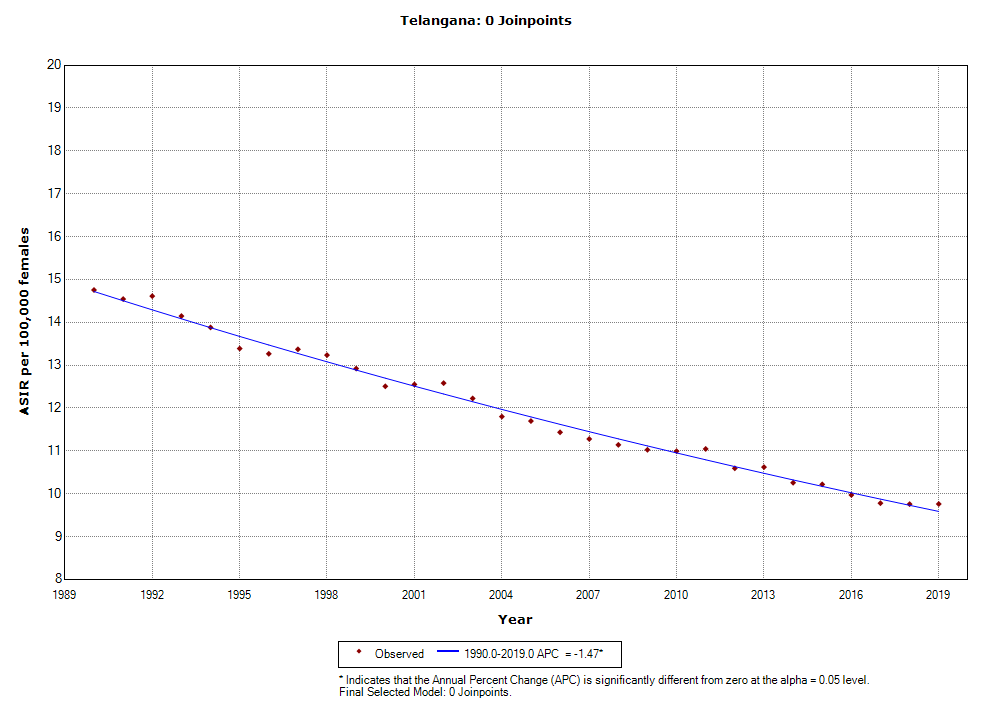 | 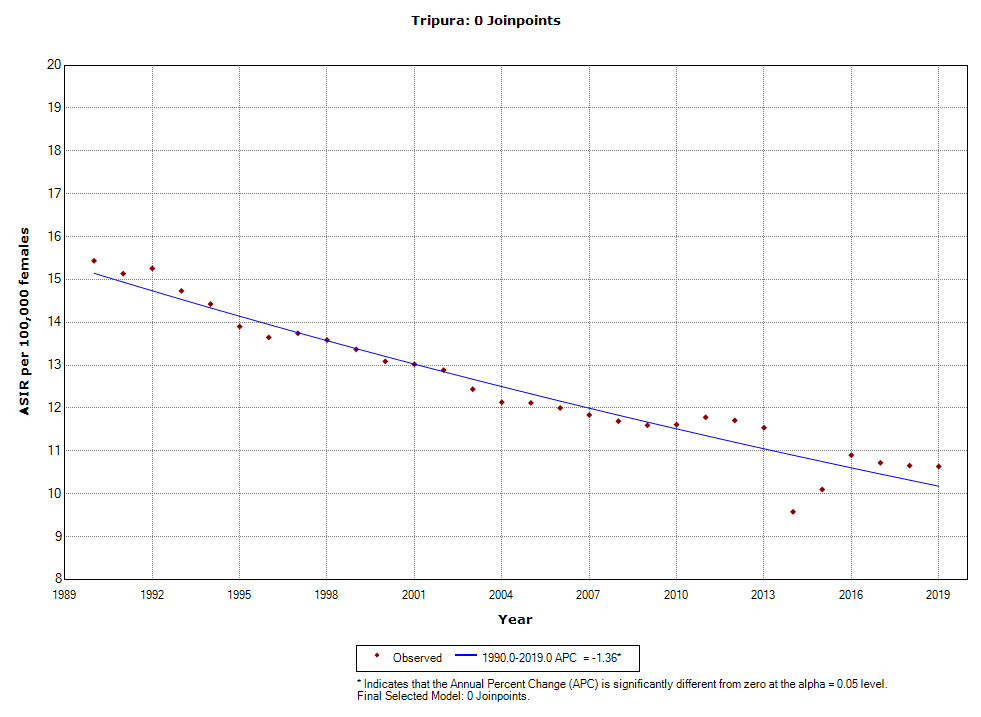 | 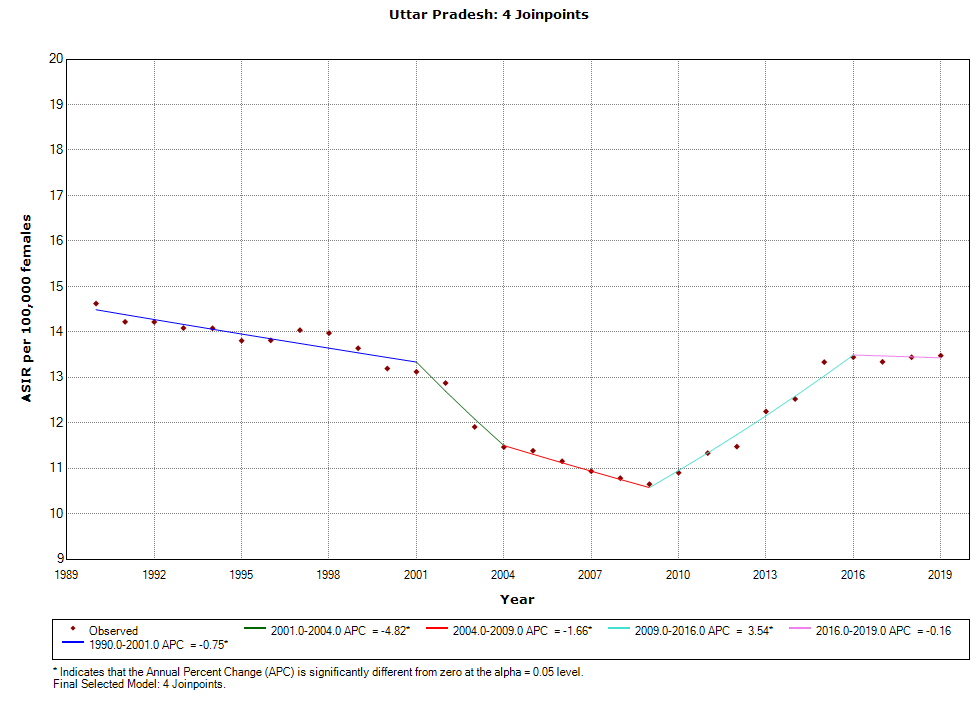 |
| 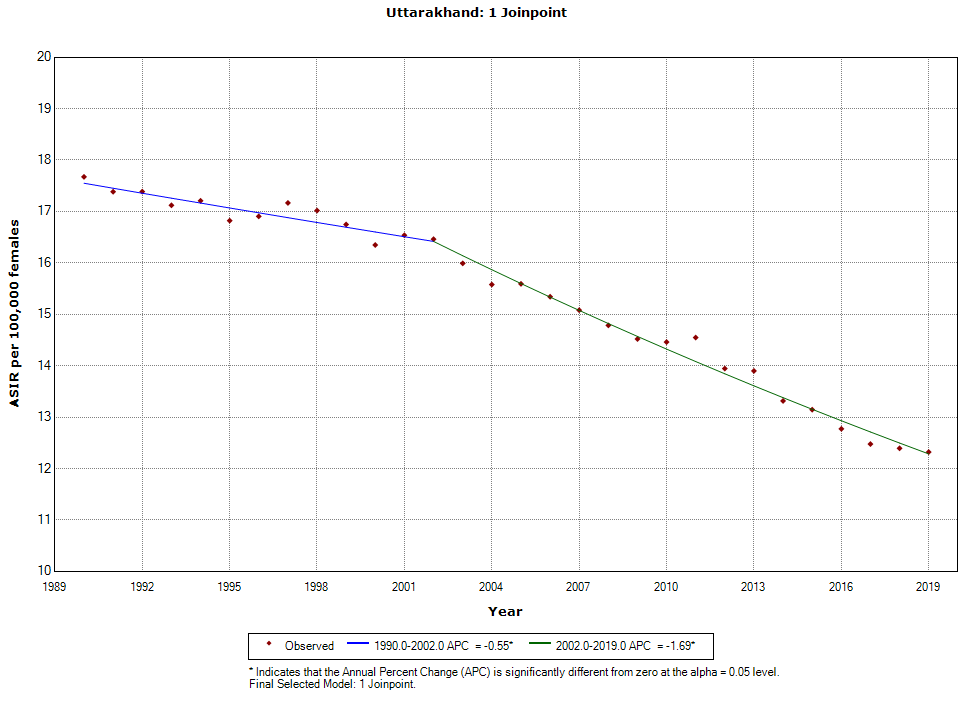 | 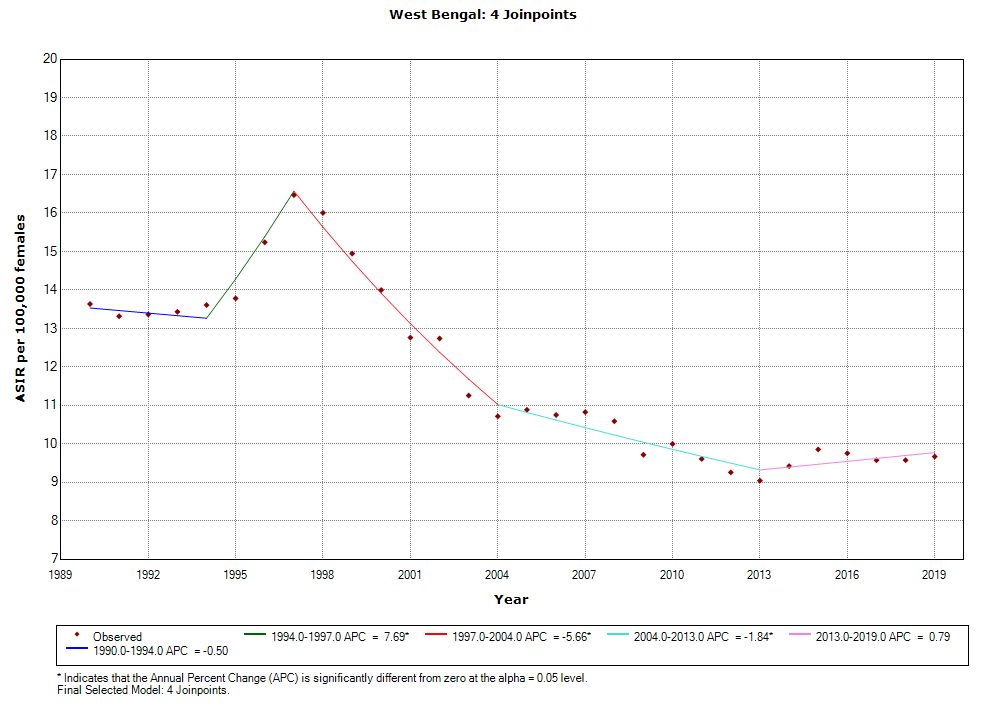 |  |

**Supplementary Figure 2: Trends in age standardized mortality rate of cervical cancer using joinpoint regression analysis across states of India**

| 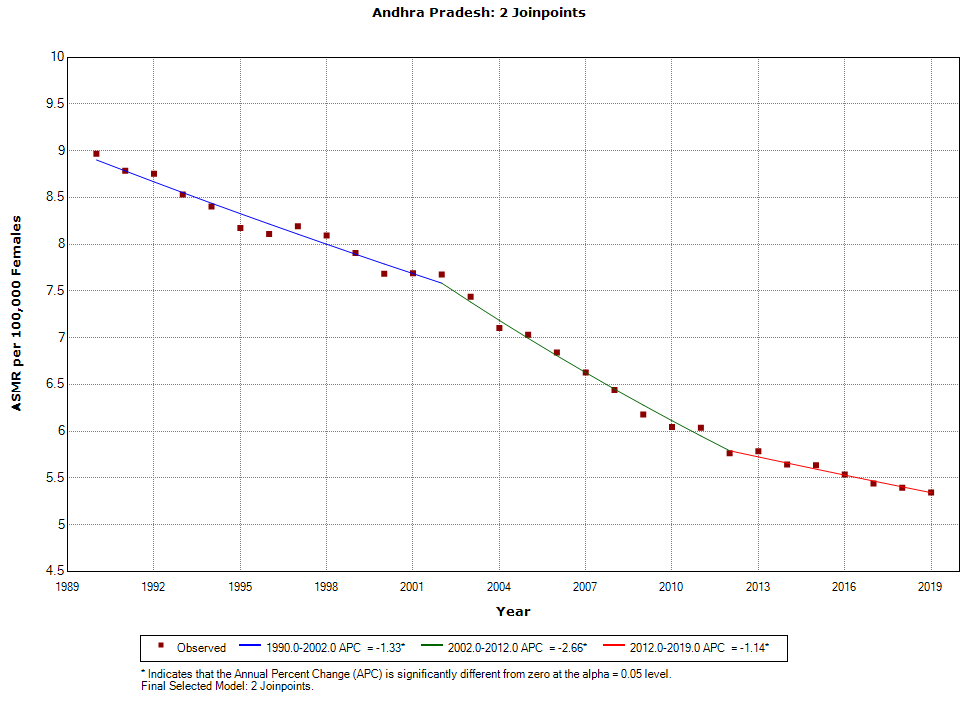 | 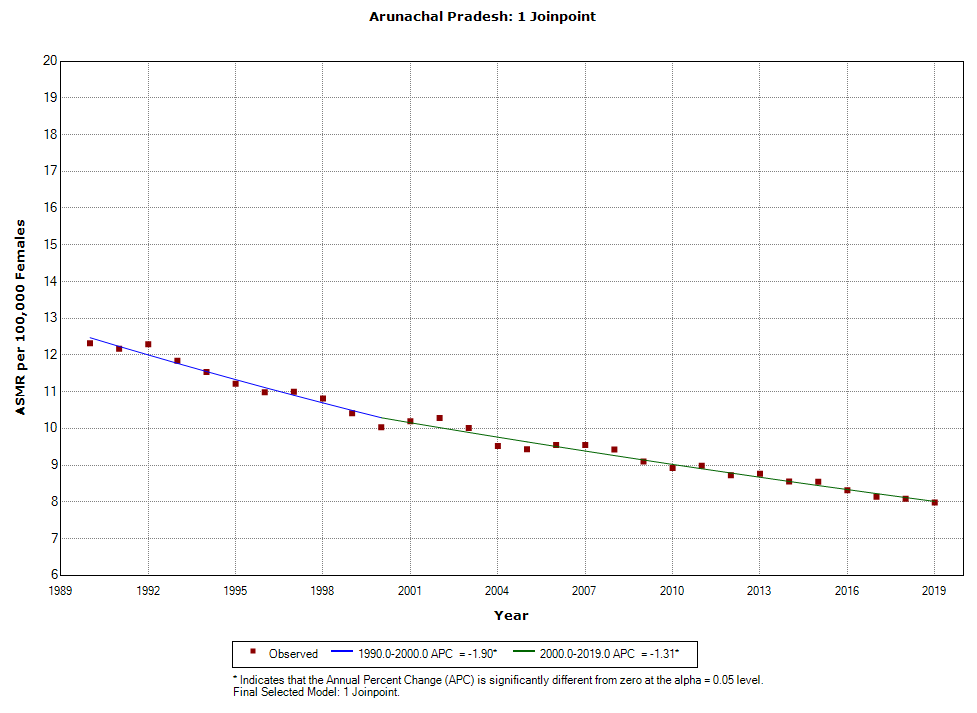 | 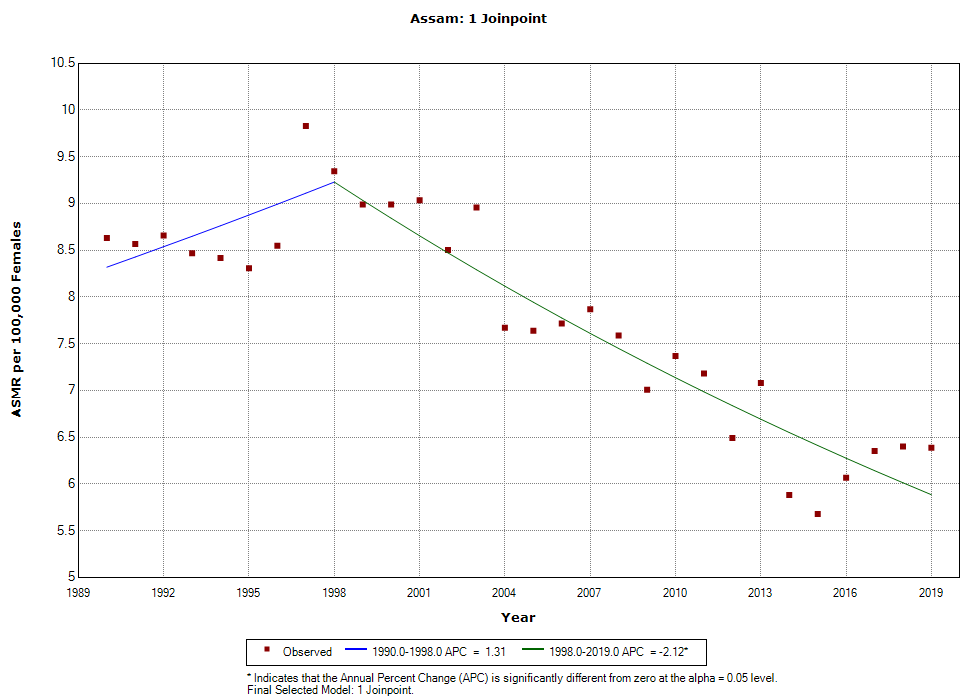 |
| --- | --- | --- |
| 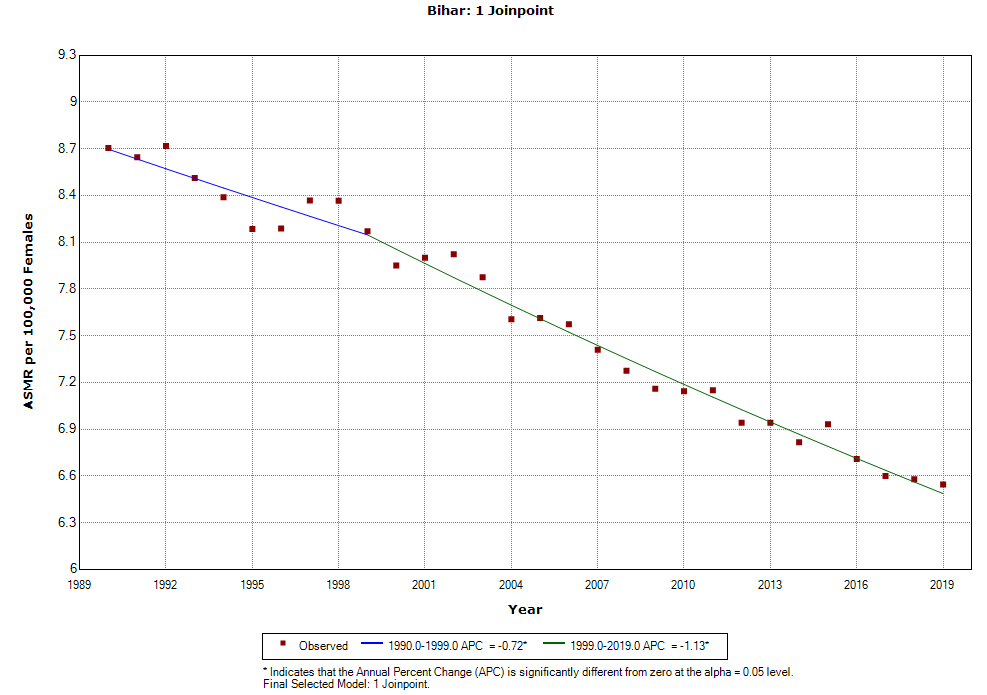 | 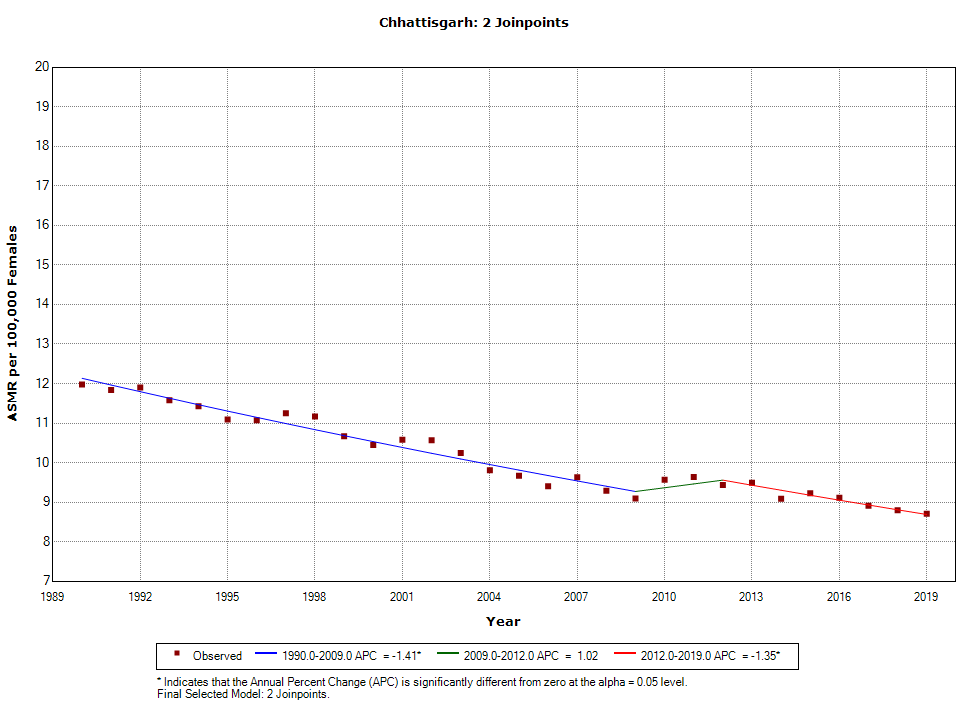 | 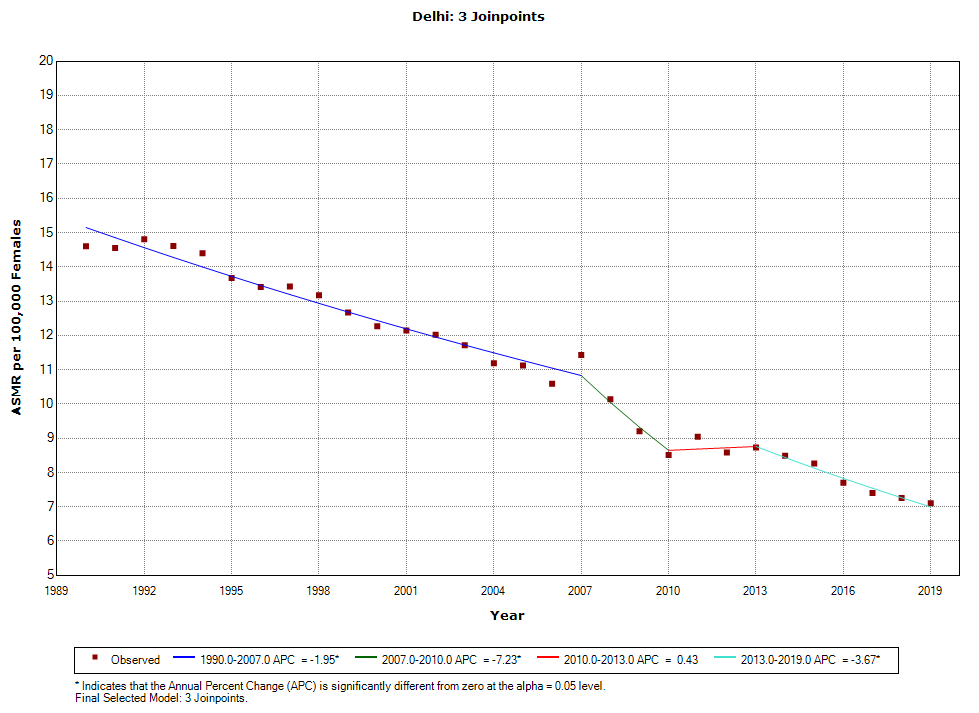 |
| 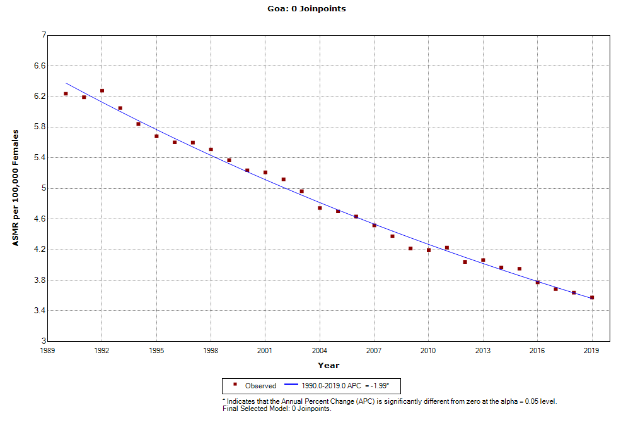 | 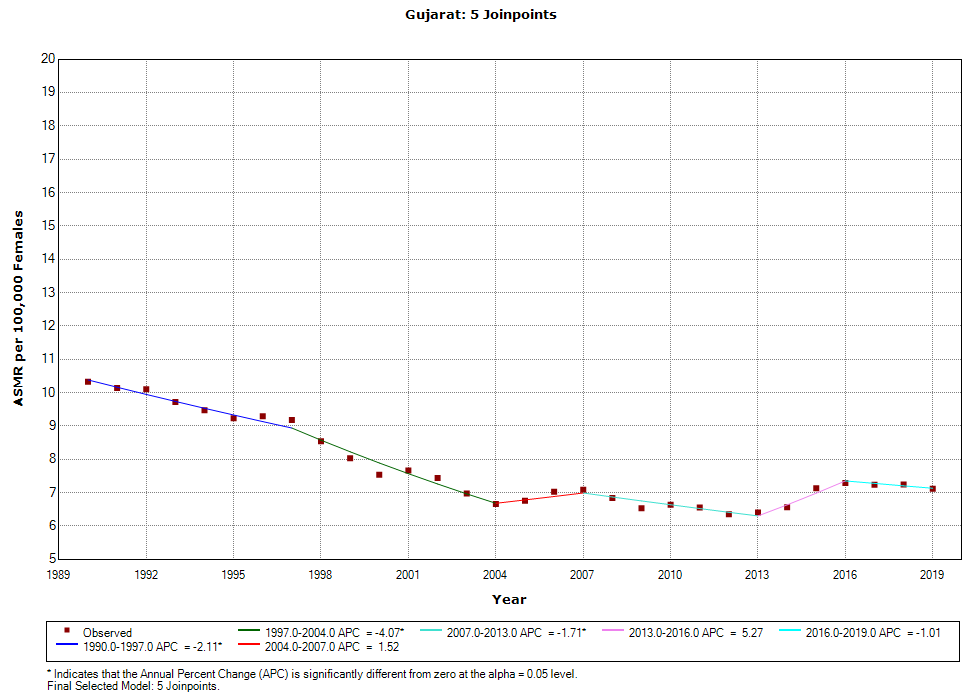 | 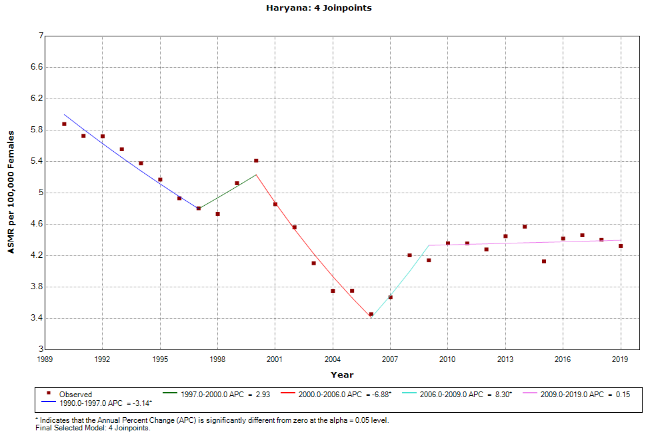 |
| 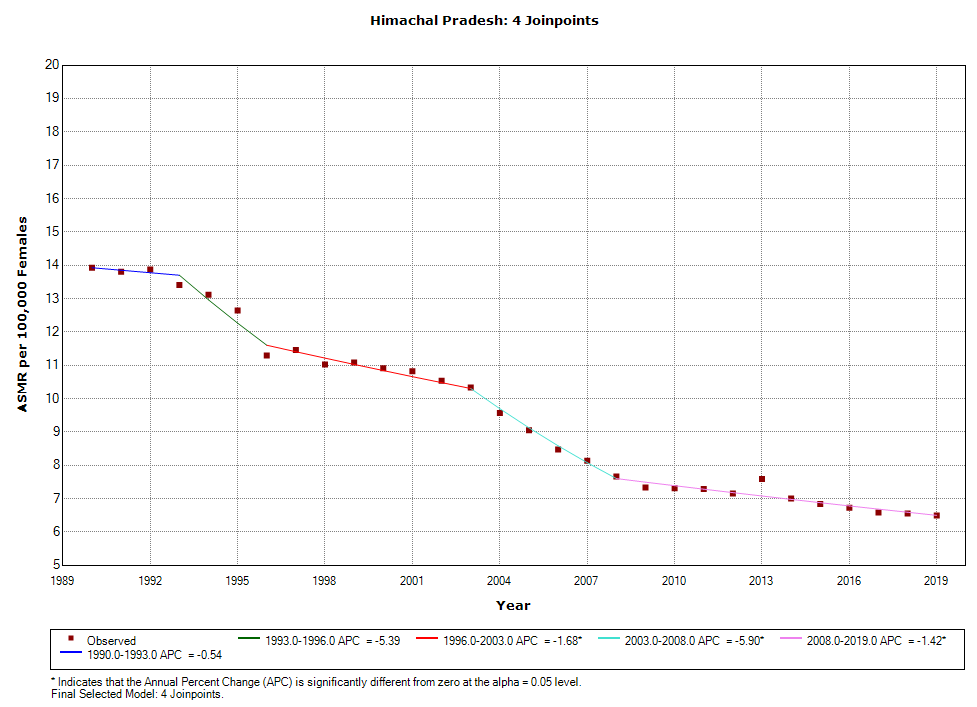 | 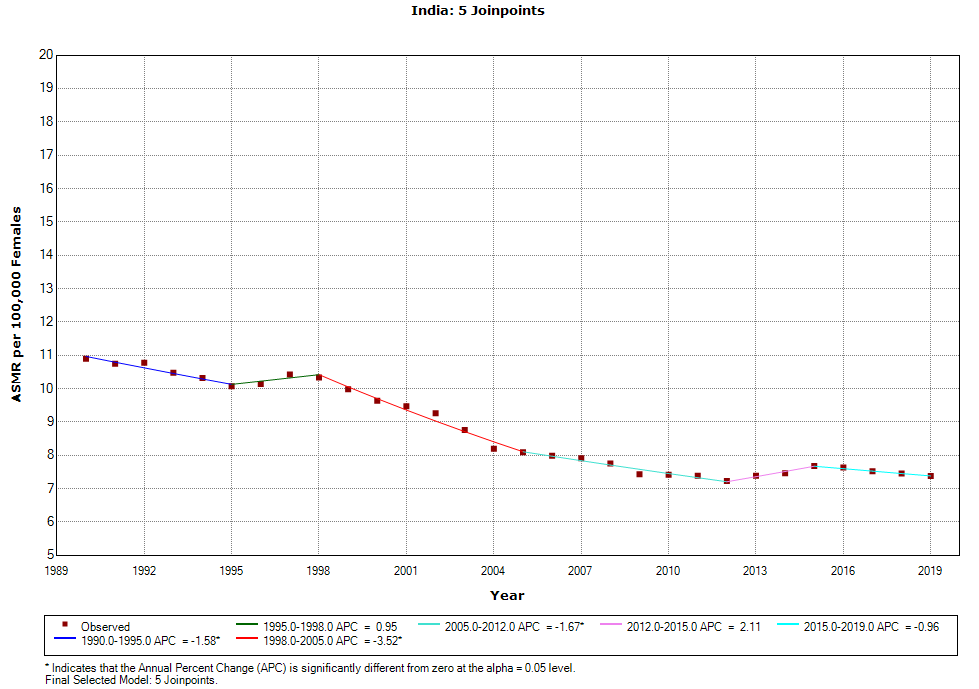 | 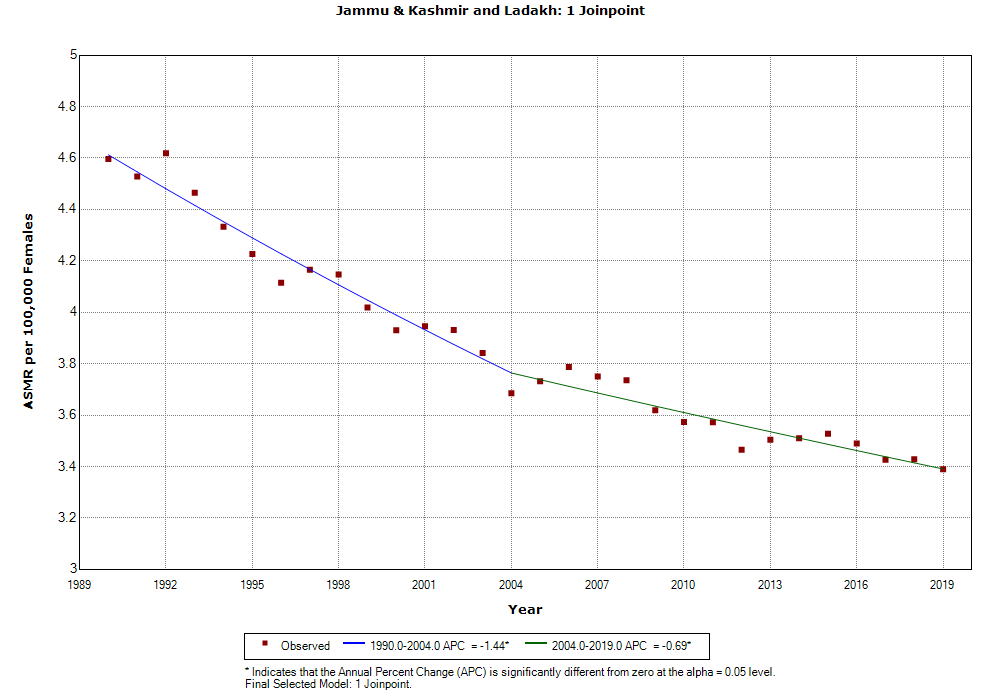 |
| 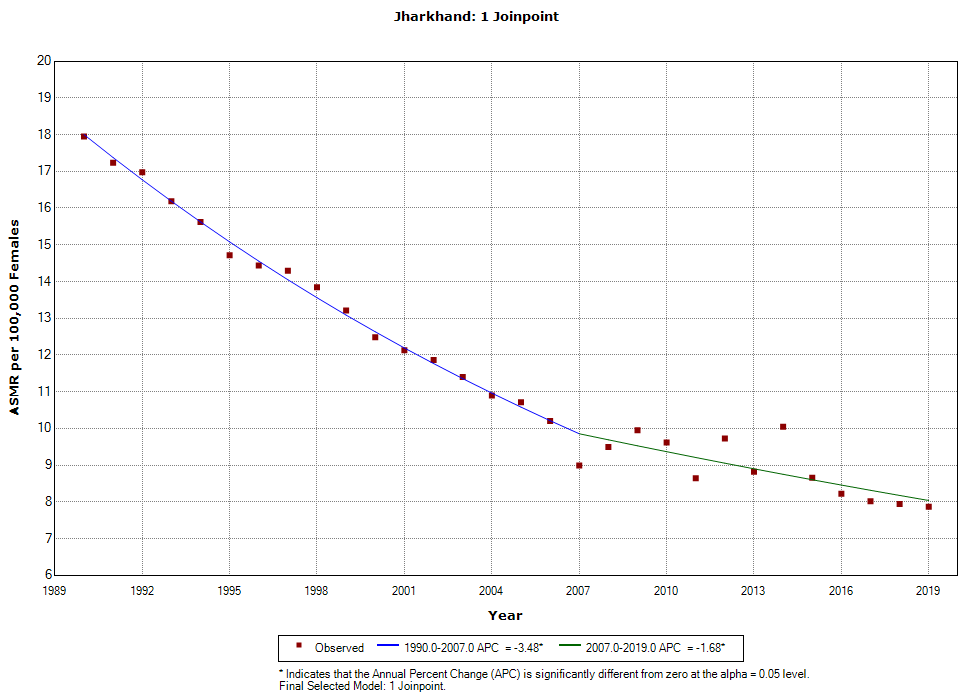 | 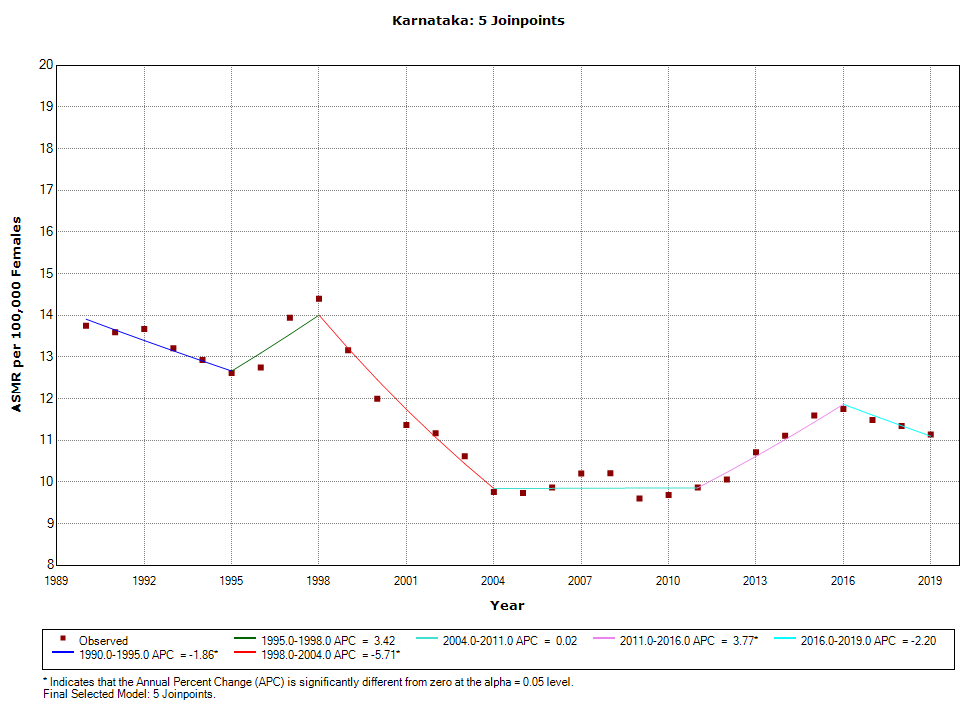 | 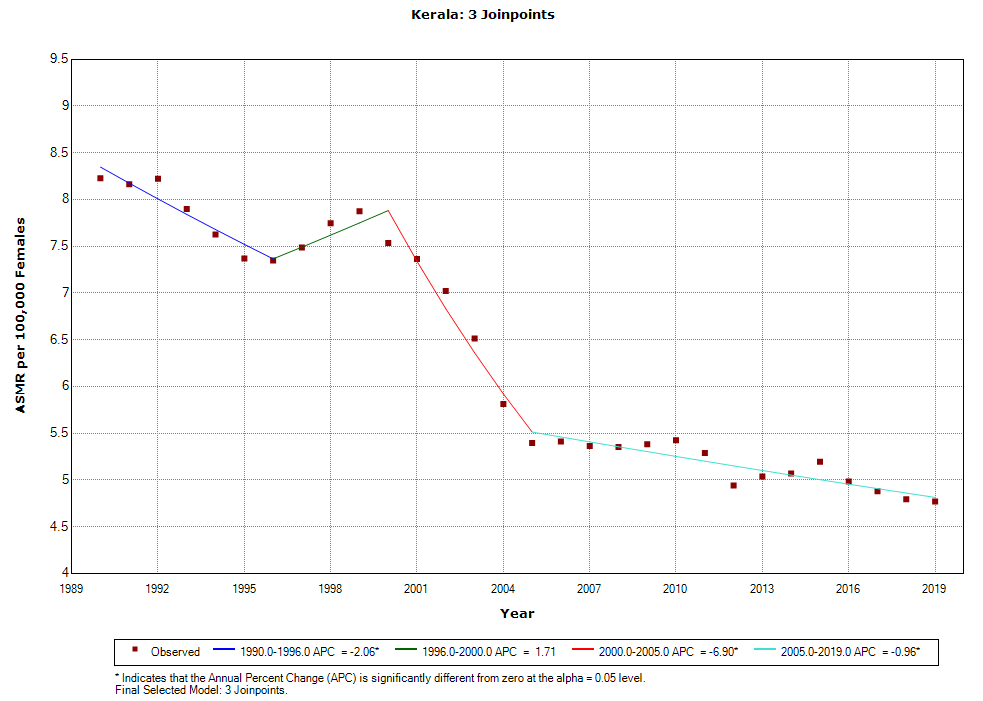 |
| 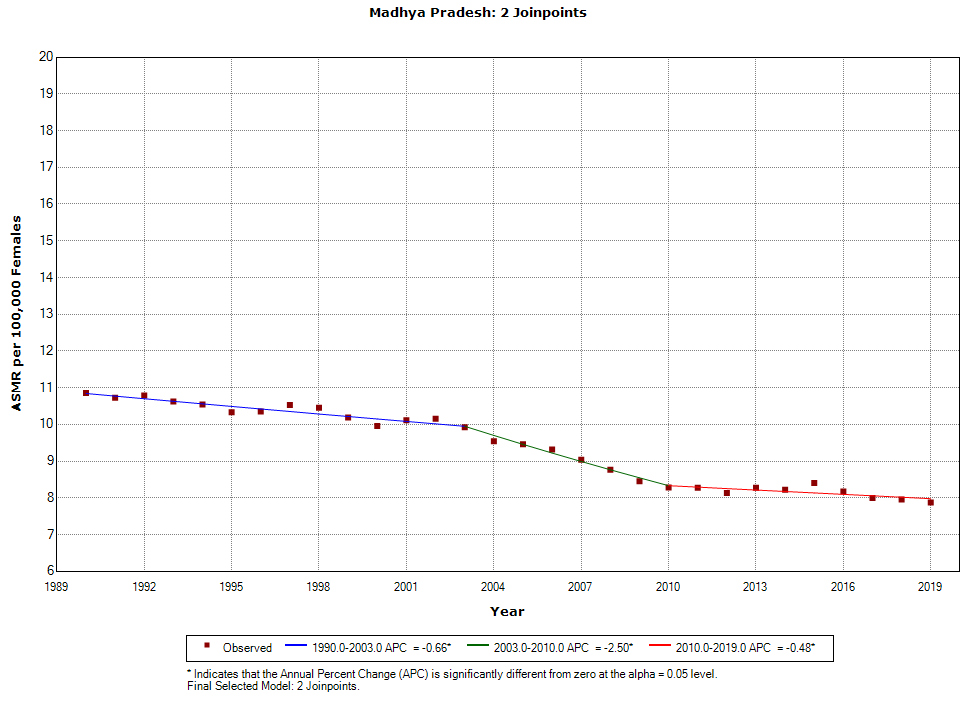 | 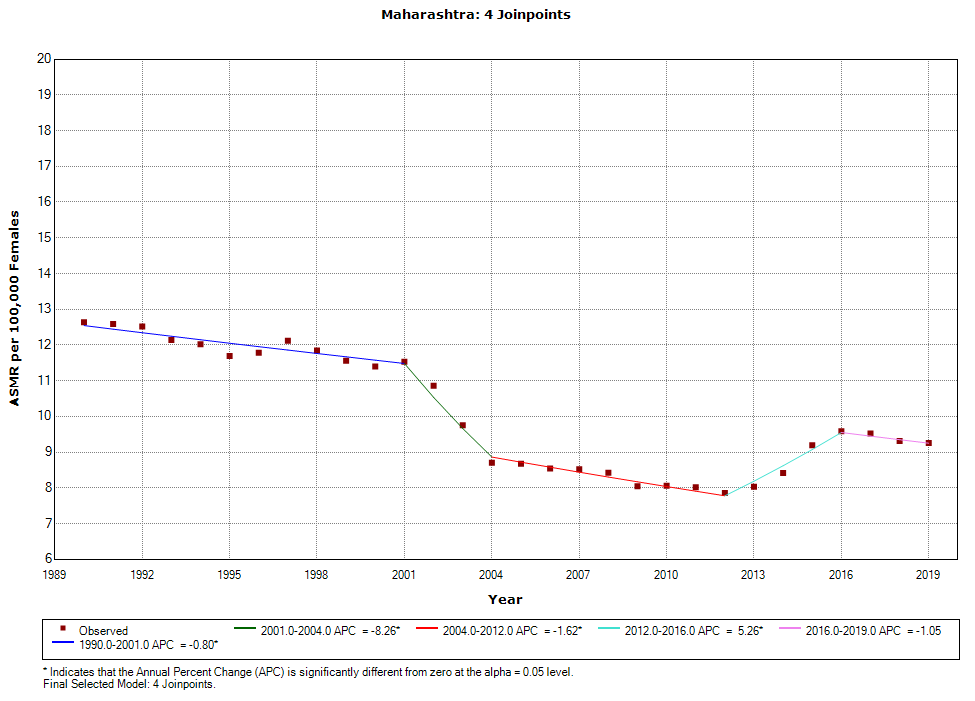 | 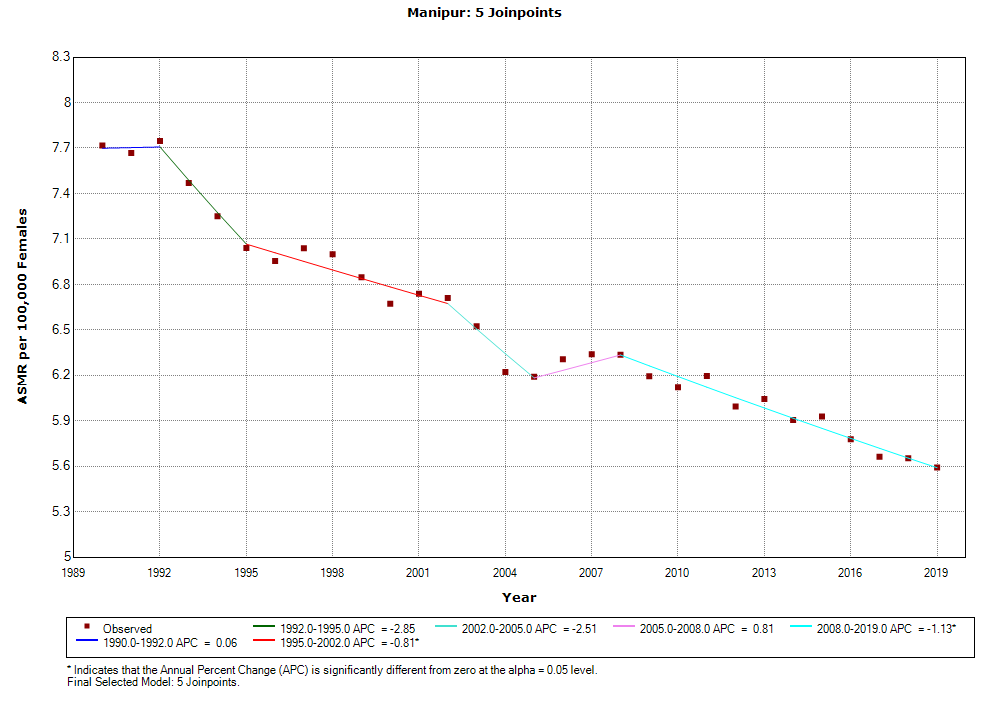 |
| 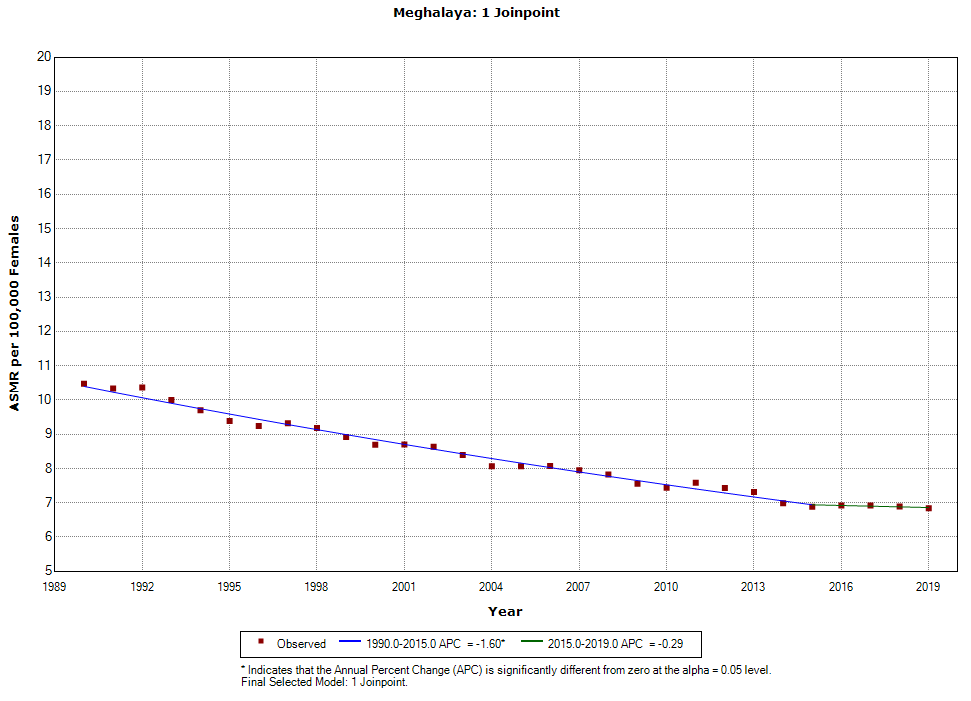 | 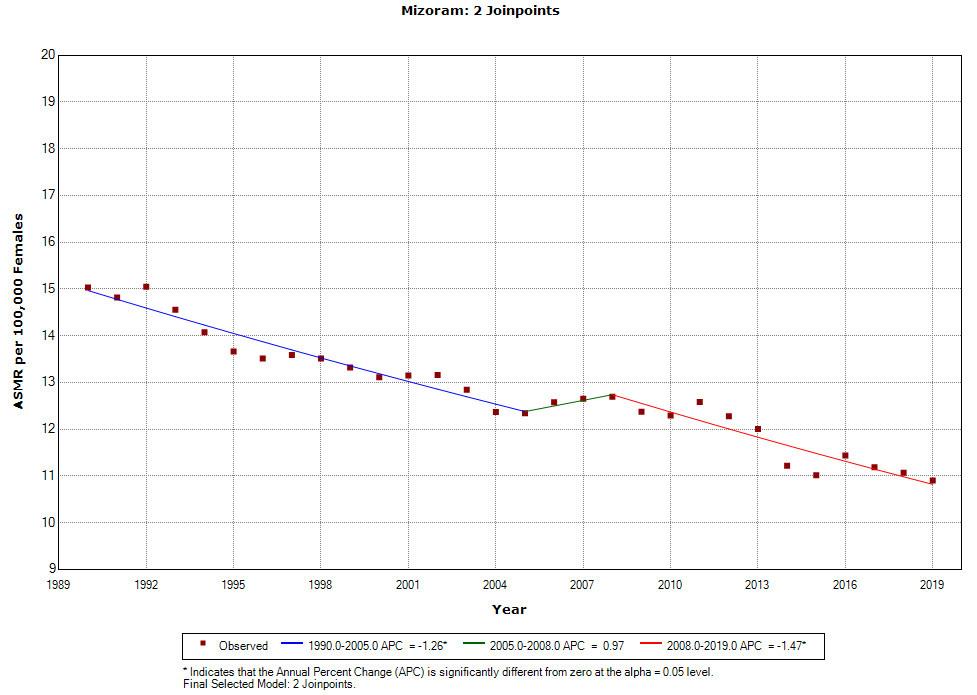 | 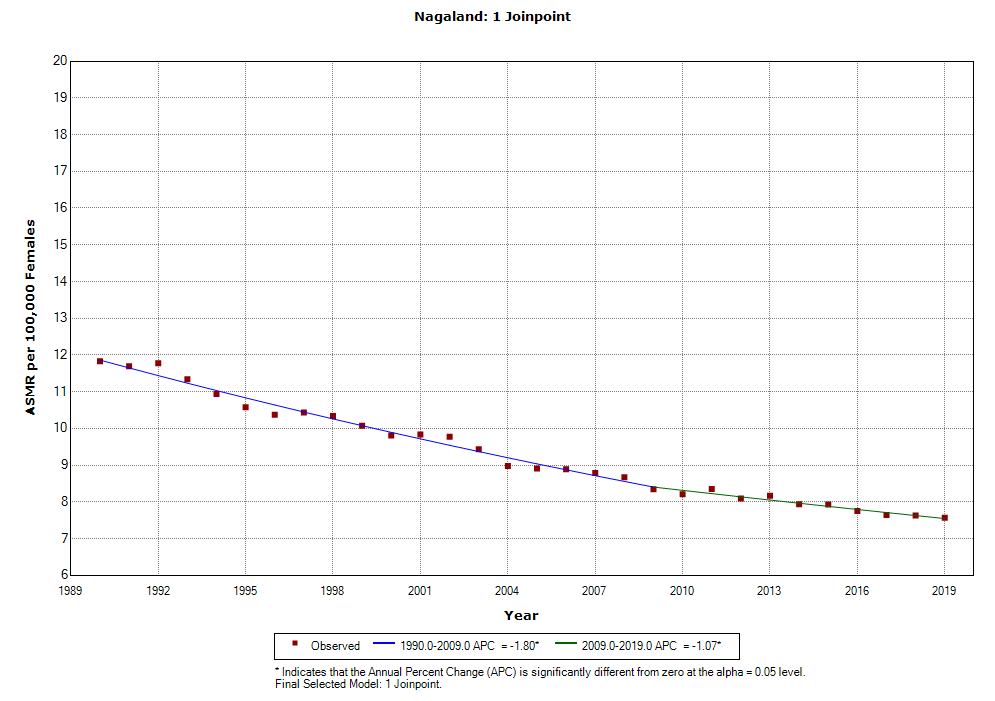 |
| 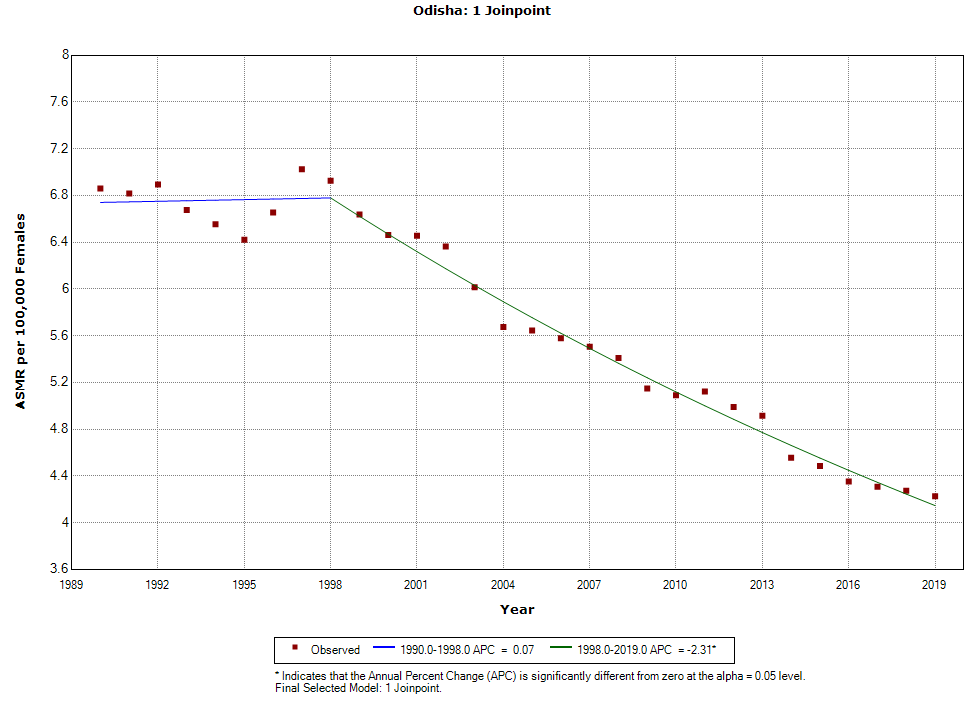 | 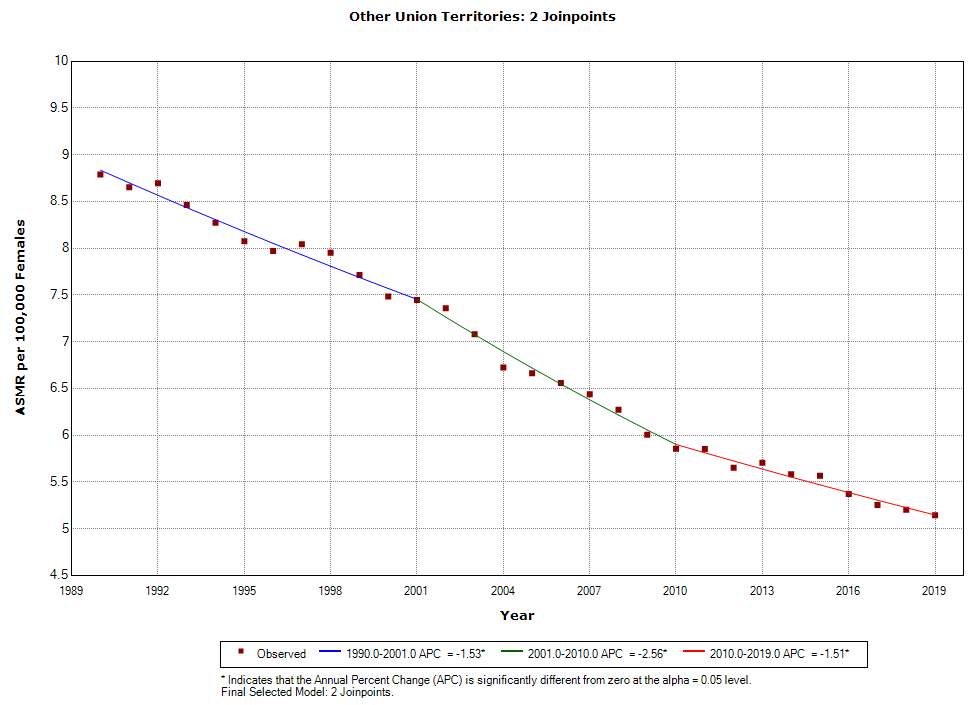 | 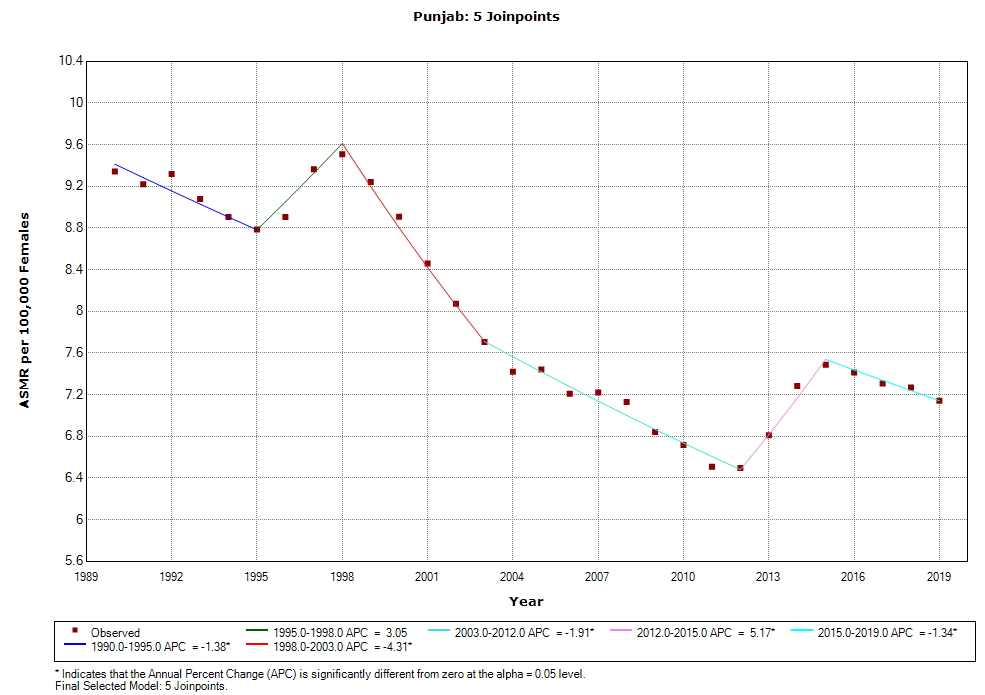 |
| 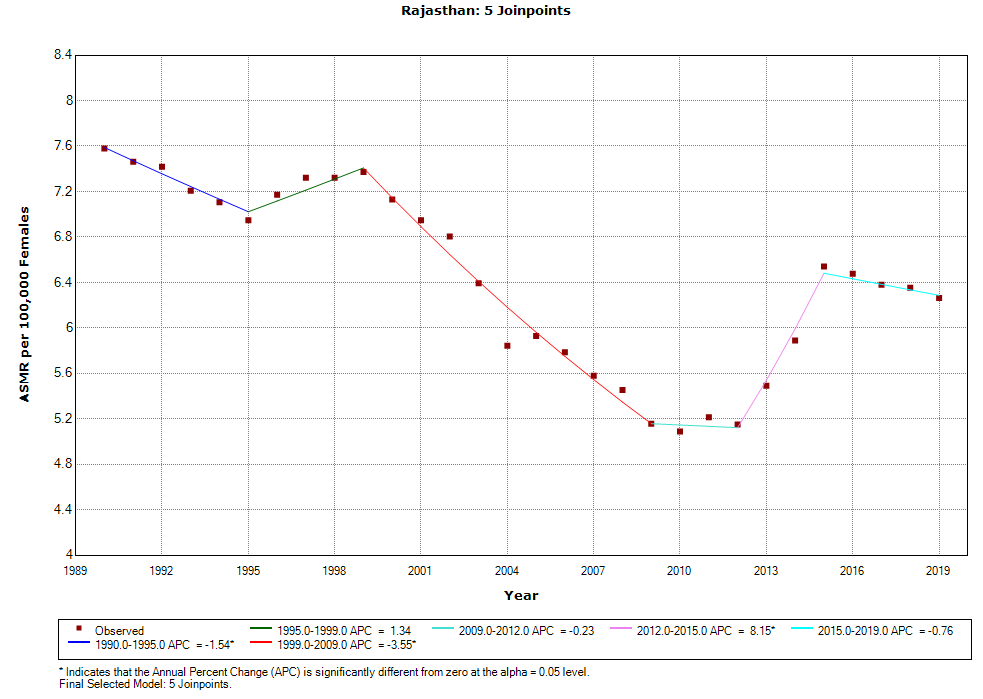 | 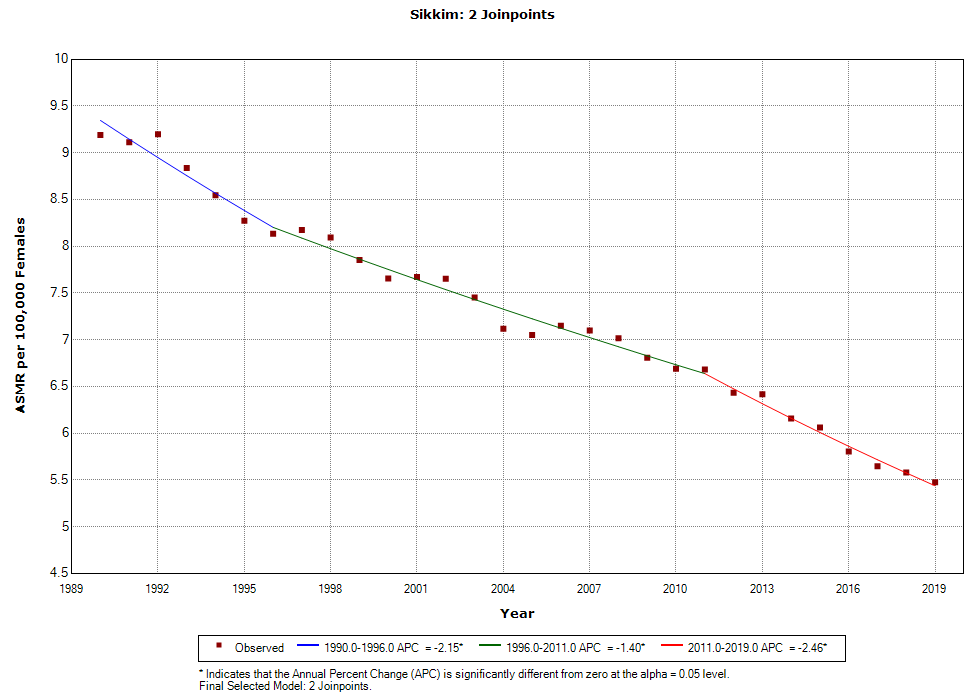 | 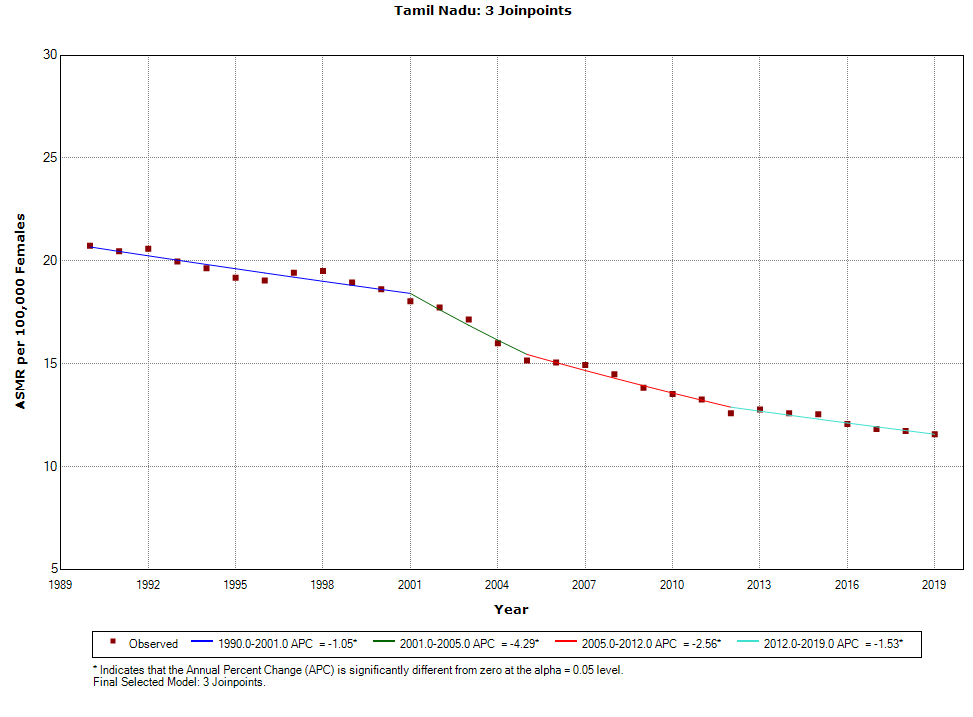 |
| 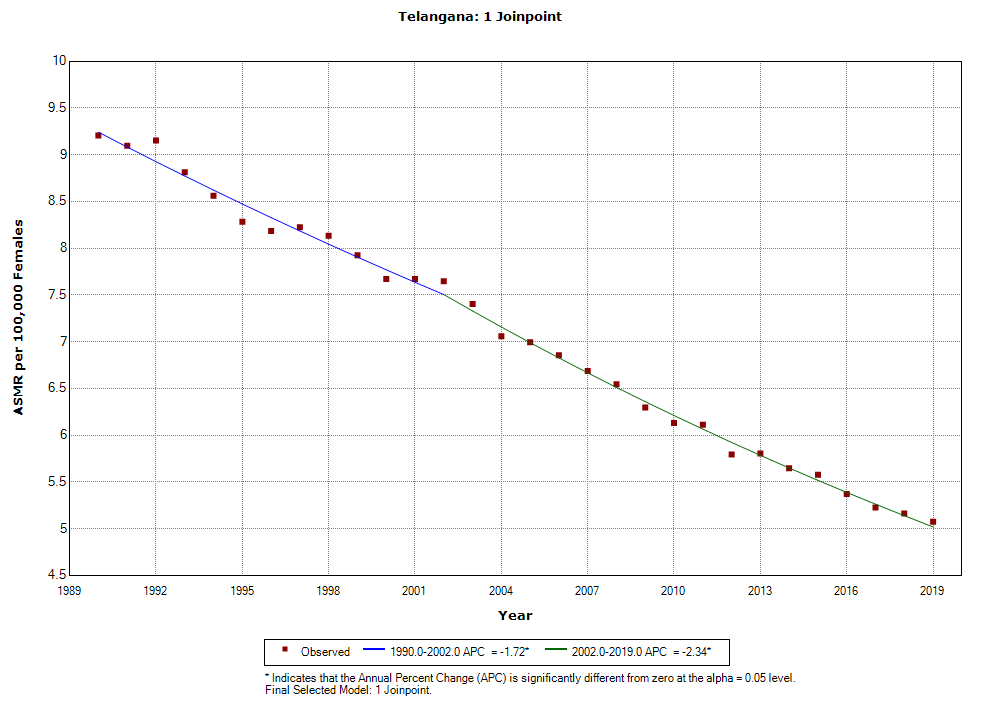 | 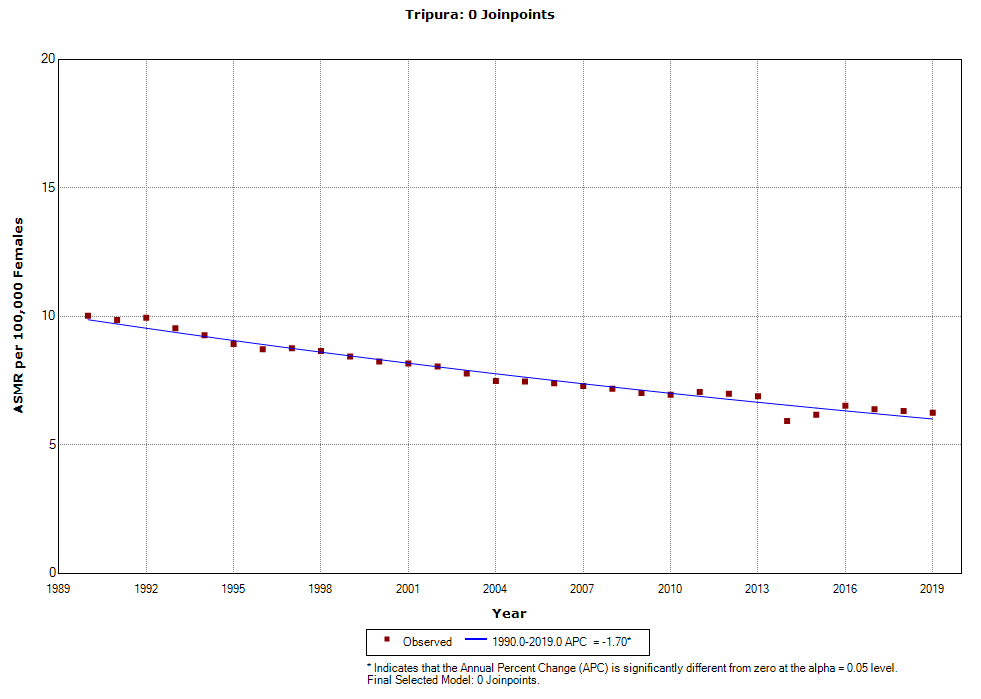 | 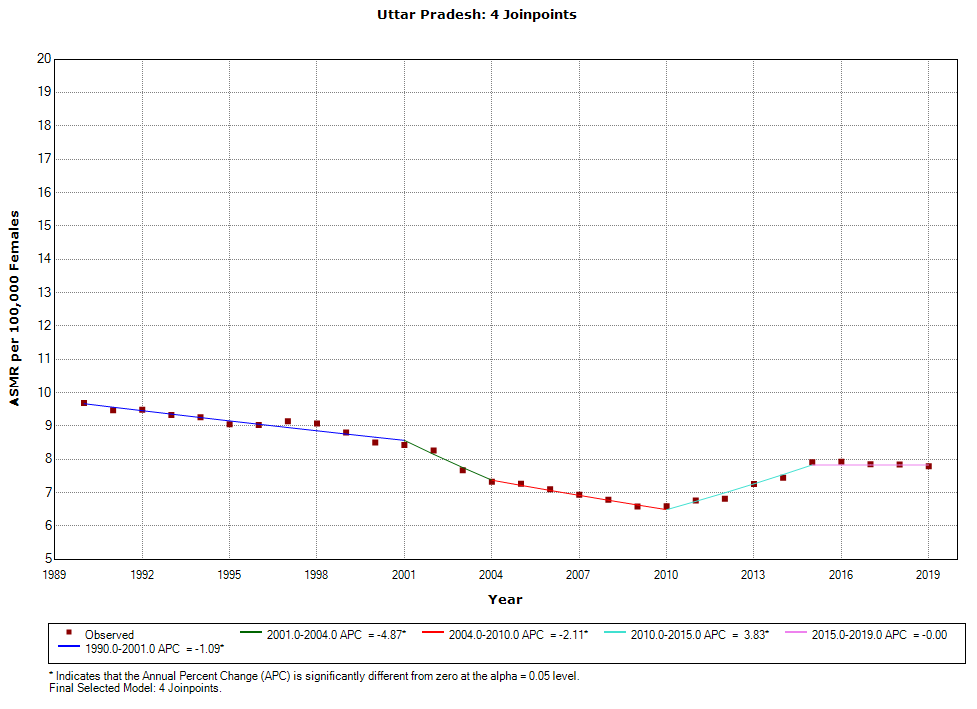 |
| 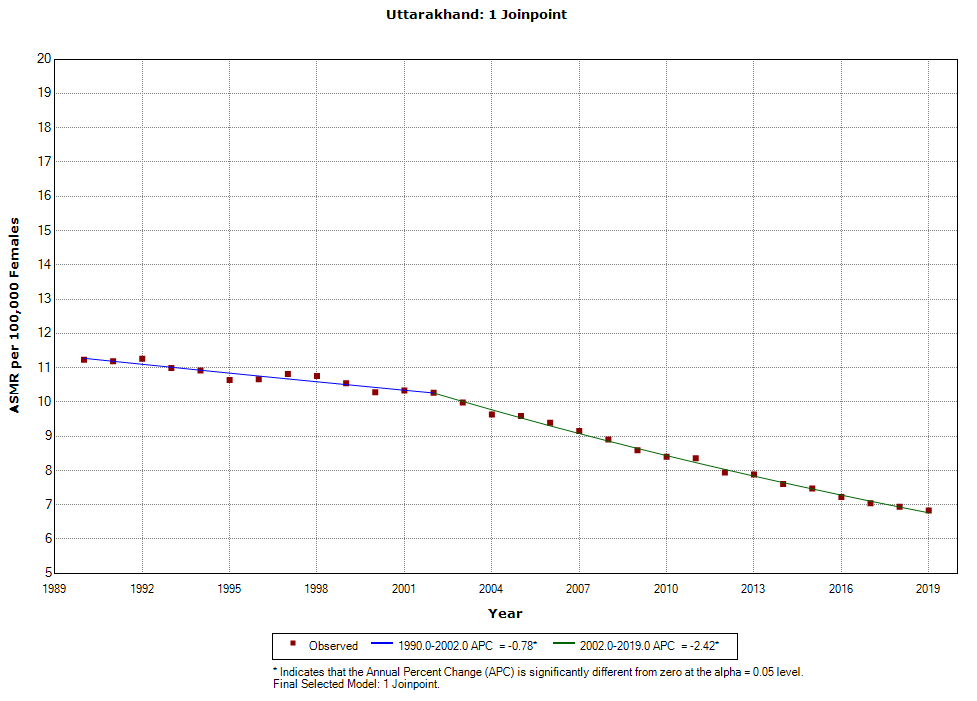 | 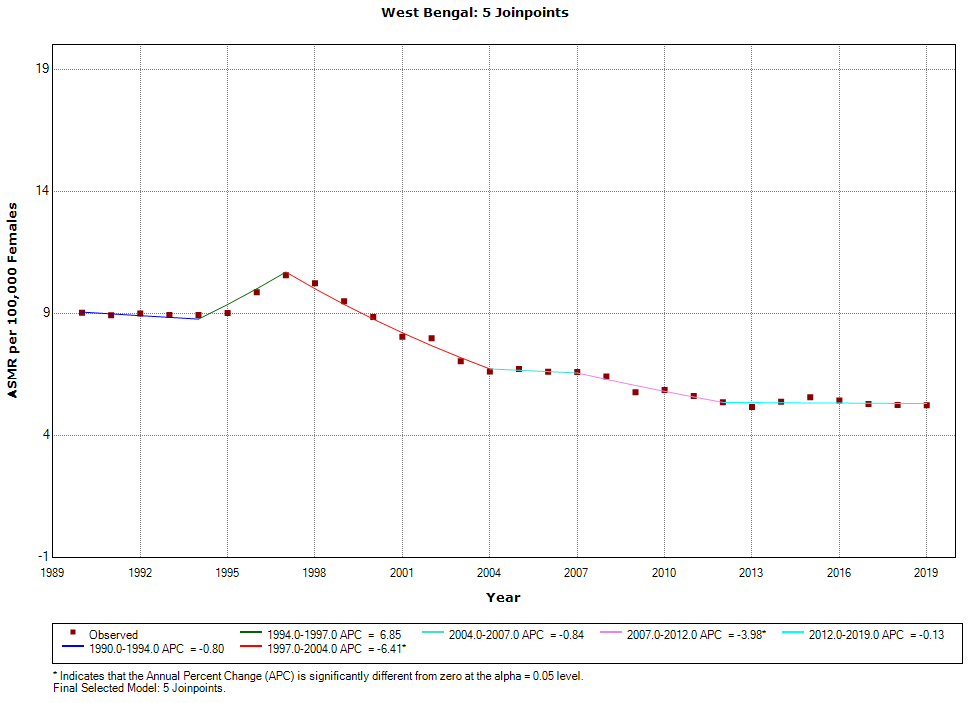 |  |

**Supplementary Figure Legends**

Supplementary Figure 1: Trends in age-standardized cervical cancer Incidence rate using joinpoint regression analysis across states of India

Supplementary Figure 2: Trends in age-standardized mortality rate of cervical cancer using joinpoint regression analysis across states of India

**Supplementary Table Legends**

Supplementary Table 1: Percentage changes in cervical cancer incidence among women of all ages in India and its states over the period 1990 to 2019.

Supplementary Table 2: Percentage changes in cervical cancer mortality among women of all ages in India and its states over the period 1990 to 2019.
